# The NONO protein regulates nonclassical DNA structure: Effects on circadian genes and DNA damage

**DOI:** 10.1016/j.isci.2025.112408

**Published:** 2025-04-11

**Authors:** Ermanno Moriggi, Melissa Pisteljic, Alex Rosi-Andersen, Lennart Opitz, Abdelhalim Azzi, Steven A. Brown

**Affiliations:** 1 Institute of Pharmacology and Toxicology, University of Zurich, Zurich, Switzerland; 2 Experimental Psychopathology and Psychotherapy, Department of Psychology, University of Zurich, Zurich, Switzerland; 3 Functional Genomic Center Zurich, ETH and University of Zurich, Zurich, Switzerland; 4 Laboratory of Lipids and Chronobiology, IMol, Polish Academy of Sciences, Warsaw, Poland

**Keywords:** Nucleic acids, Properties of biomolecules, Molecular mechanism of gene regulation, Molecular interaction, Transcriptomics

## Abstract

The DBHS protein family of Nono, PSPC1, and SFPQ regulates diverse aspects of RNA metabolism. Whether these proteins share similar functions is currently unknown. In mouse embryonic fibroblasts (MEFs), we observed around 2000 circadian and non-circadian genes regulated by Nono and PSPC1, with only 35% in common. Considering specifically circadian genes, up- or downregulation by Nono and PSPC1 depends mainly on the gene phase. We postulated a regulatory role of Nono on R-loops, the class of non-B DNA structures that form during transcription. We confirmed this by showing a broad effect of Nono on genome-wide R-loop homeostasis. Interestingly, the R-loop regulation by Nono occurs in a time-of-day dependent manner among the circadian genes. Moreover, we showed a protective role of Nono in a DNA damage cellular model that involves R-loop accumulation. Further studies are required to understand the circadian regulation of R-loops and their implications on gene regulation and disease.

## Highlights

• Nono and PSPC1 regulate a common set of circadian genes in a phase-dependent manner
• Nono has a broad effect on genome-wide R-loop homeostasis
• The R-loops at the promoter of circadian genes show circadian dynamics
• Nono has a protective role at DNA damage sites through R-loops modulation

## Graphical abstract

**Figure:**
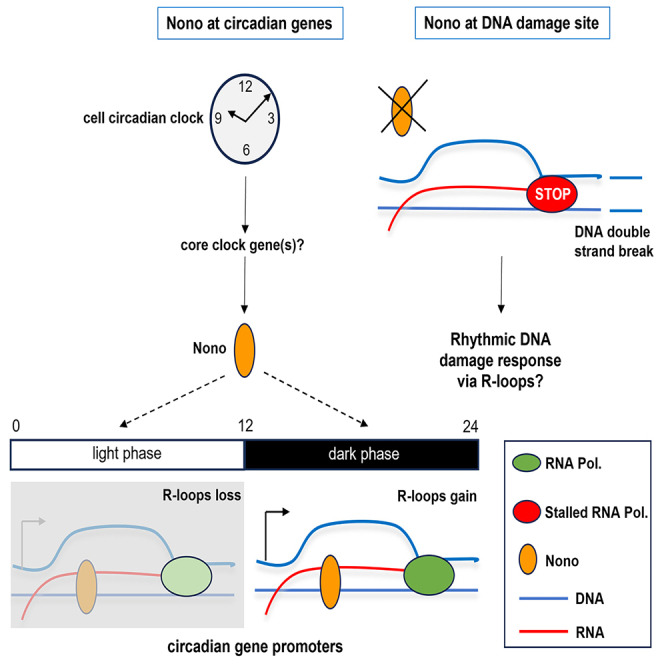

## Introduction

The Non-POU Domain-containing Octamer Binding Protein (Nono) – also known as p54nrb in humans and NonA in fruit flies – belongs to the Drosophila Behavior Human Splicing family of proteins (DBHS) and has been implicated in the circadian clock metabolism, brain function, and cancer.^1^^,^^2^^,^^3^^,^^4^ Nono is mainly located in the nucleus and, together with its DBHS partners, it is involved in many steps of RNA metabolism, including transcriptional activation and inhibition, RNA binding and processing, retention of edited RNAs in nuclear paraspeckles, RNA transport, and DNA repair.^5^ Interestingly, Nono has been described as both a coactivator and corepressor of transcription. Indeed, Nono binding to androgen receptors promotes their transcriptional activity. Moreover, Nono promotes the cAMP-dependent activation of CREB target genes, whereas the NONO-mediated recruitment of mSin3A to the progesterone receptors alters their transcriptional activity.^6^^,^^7^^,^^8^

The common domains of DBHS proteins (two highly conserved tandem N-terminal RNA recognition motifs (RRMs), a NonA/paraspeckle domain (NOPS), and a nuclear localization signal containing C-terminal coiled-coil) explain their functional overlap and, in some cases, the compensation for the loss of one of them. However, the unique sequences at the N- and C-terminal, and the fact that they seem to work as obligatory dimers, open the possibility of distinct roles for each protein and of different DBHS complexes. An example of the lack of compensation is the Nono regulation of synaptic transcription in a complex with either SFPQ or PSPC1; in this case, the lack of Nono results in intellectual disability.^3^ Of particular interest is the role of DBHS proteins in the regulation of circadian clock outputs. Indeed, it has been shown that all three DBHS proteins are implicated in the transcriptional regulation of the circadian clock.^9^

For example, Nono binds and modulates levels of PER proteins, which play an essential role in the negative transcriptional feedback loop of the molecular clock. Moreover, NONO binds the Rev-Erbα promoter in a circadian fashion.^9^ In addition, it has been demonstrated that Nono determines a circadian “pause” in the cell cycle progression and couples the clock to the cell cycle upon direct binding to the promoter of the cell cycle inhibitor p16-INK4A.^1^

A further role was recently demonstrated for Nono in the circadian gene expression and physiology in the liver.^2^ By pre-mRNA processing of certain glucose-responsive genes, Nono controls their rhythmicity and phase, finally resulting in a switch from glycogen use to fat oxidation because of the inability of the liver to properly store glycogen. Together, these findings show that Nono can modulate several processes through its protein-protein interaction or RNA-interaction modules.

In this respect, several recent proteomic studies reported that DBHS proteins, particularly Nono, are part of the R-loops interactome.^10^^,^^11^^,^^12^ R-loops are structures that form during transcription and are composed of a complementary RNA/DNA hybrid and the displaced non-template DNA strand. R-loops have been described as important regulators of many cellular processes. They participate in gene regulation, favoring or silencing transcription at the promoter, in RNA processing, and they seem necessary for correct transcription termination.^13^^,^^14^ In addition to regulatory R-loops, the existence of unscheduled R-loops has been suggested, which can have detrimental effects on transcription, DNA damage, and genome stability.^15^^,^^16^^,^^17^ In fact, they are thought to contribute to pathologies and diseases, such as neurodegenerative disorders and cancers.^18^^,^^19^

R-loop number and distribution are very dynamic and carefully controlled by different cell systems, and new R-loop modulators are often proposed as well.^14^^,^^20^ Interestingly, Nono/SFPQ heterodimers have been described to promote telomer integrity by resolving the RNA/DNA hybrids.^21^ Furthermore, Nono alone or in combination with SFPQ has been demonstrated to play a role in DNA repair and promote genome maintenance pathways such as cell response to UV and DNA double-strand break (DSB) repair via non-homologous end-joining.^22^^,^^23^ In addition, Nono has been shown to be involved in the DNA Damage Response (DDR).^24^^,^^25^ Therefore, we speculated that the ability of Nono to influence R-loops dynamics extended much beyond the telomeres and aimed to investigate it genome-wide. To gain insights into how NONO regulates mRNA life and R-loop dynamics, we carried out detailed steady-state transcription and genome-wide profiling of DNA/RNA hybrids. Herein, we highlighted the Nono regulation of RNA synthesis and alternative splicing, depending on the circadian time. Furthermore, we showed that Nono depletion alters genome-wide R-loop homeostasis, which correlates with circadian gene expressions and DNA damage. Overall, our work unveiled new functions of Nono in R-loop regulation.

## Results

### Nono and PSPC1 control global circadian transcription

Nono has been suggested to mechanistically regulate diverse aspects of RNA metabolism in very heterogeneous ways.^26^^,^^27^^,^^28^ However, the Nono genome-wide mRNA regulation is still poorly defined.

We started by precisely defining the circadian transcriptome in mouse embryonic fibroblasts (MEFs). RNA-seq analysis was carried out on purified RNA samples from WT cells collected at intervals of 4 h after the synchronization of the molecular clock by Dexamethasone exposure (6 time points; samples were analyzed in triplicate). Evidence of the effective synchronization is shown in Figure S1.

Periodogram analysis indicated a dominant period of 24 h (Figure S2A). During this period, two commonly used approaches for oscillation detection, the Cosinor, and JTK-Cycle, were used to determine circadian rhythmic gene expression.^29^^,^^30^

**Figure 1:**
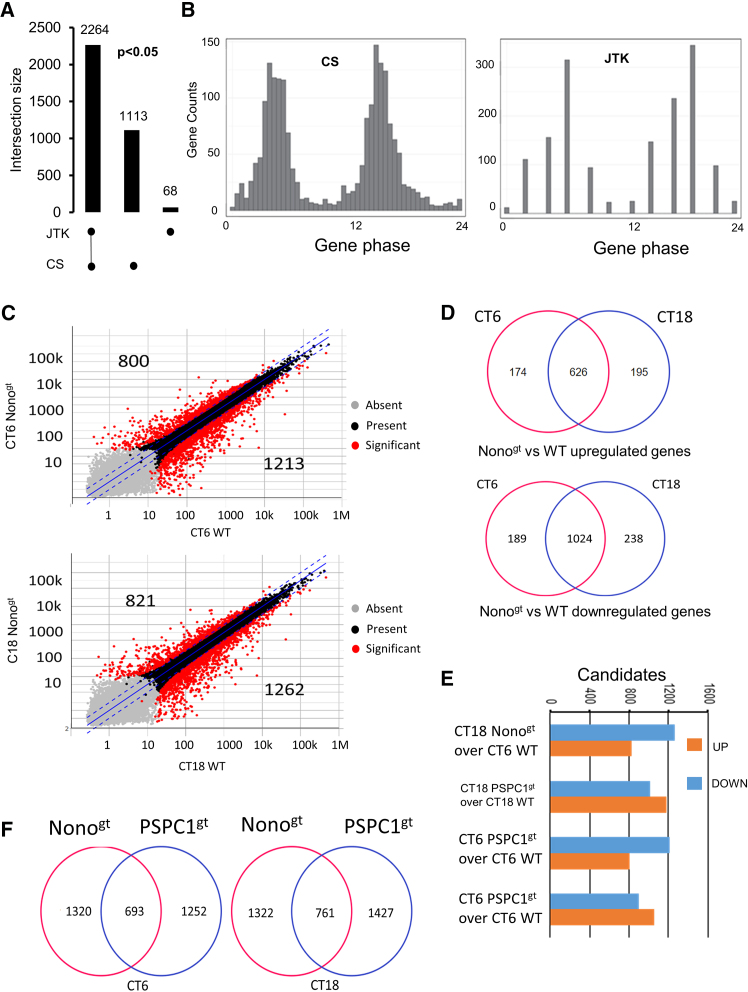
Nono and PSPC1 depletion alters global circadian transcription in MEFs (A) Bar graph showing the overlapping and unique number of circadian genes identified by Cosinor and JTK Cycle analyses in MEFs (*p* < 0.05). (B) Column graphs showing the two distinct acrophase peaks of circadian genes obtained with Cosinor and JTK Cycle. The acrophases are in the range 4–6 h and 16–18 h. (C) Scatterplots representing color-coded expression levels of differentially expressed genes (DEG) in Nono^gt^ vs. WT (edgeR, FDR<0.01, |log2FC|>1) at the indicated time points. (D) Venn diagram showing the extent of overlap of the DEGs in WT and Nono depleted cells analyzed at the indicated time points (Fisher’s exact test: Upregulated genes: *p*-value <2.2e-16, odds ratio 224.2; Downregulated genes: *p*-value <2.2e-16, odds ratio 269.1). (E) Bar graph showing the results of the two-group analysis of up- and downregulated genes by Nono and PSPC1, at the two time points corresponding to the phase peaks of circadian genes (edgeR, FDR<0.01, |log2FC|>1). (F) Venn diagram showing the extent of overlap of the DEG obtained in Nono and PSPC1-depleted cells vs. WT, analyzed at the indicated time points (Fisher’s exact test: CT6 *p*-value <2.2 e−16, odds ratio 4.1; CT18 *p*-value <2.2e-16, odds ratio 3.9).

Our data showed that 18% of the expressed genes (2264 out of 12410) exhibited circadian rhythmicity in both analyses (Figures 1A and S2B), including core clock genes such as Arntl, Cry1, and Cry2, which are known to be essential for rhythmic gene expression (Figure S2C). It must be noted that the DBHS genes did not show RNA circadian expression. Interestingly, the circadian gene phase revealed two peaks in expression, occurring at circadian time (CT) 4–6 h and 14–18 h after cell synchronization (Figure 1B).

Gene Ontology (GO) analysis of circadian genes is shown in Figure S2D.

We next investigated how Nono and PSPC1 proteins shape the steady-state whole transcriptome of MEFs. SFPQ was not included in the investigation. In fact, SFPQ mice that are homozygous for a null allele in the whole body die embryonically, and our attempts to obtain SFPQ^gt^ MEFs only resulted in heterozygous cells.^31^

We evaluated gene expression profiles of Nono- and PSPC1-deficient MEFs (Nono^gt^ and PSPC1^gt^, respectively) compared to those of WT controls at the two time points corresponding to the phase peaks of circadian genes (18 h and 30 h after cell synchronization, named here CT18 and CT6, respectively; i.e., circadian times 18 and 6).

A comparison of the Nono^gt^ and WT MEFs revealed that 2013 and 2083 genes were differentially expressed (DEGs) at CT6 and CT18, respectively (DeSeq analysis, Log2FC ≥ 1, FDR value ≤ 0.01) (Figure 1C). Interestingly, we detected a greater number of downregulated genes in the Nono^gt^ group at both time points, suggesting that Nono plays a net co-activating role in gene expression (Figure 1C).

Moreover, the high overlap of transcriptome modifications between CT6 and CT18 in Nono^gt^ vs. WT cells indicates that Nono has a positive and negative impact on gene expression regardless of the time of day (Figure 1D).

According to the results of the two-group analysis, a similar number of differentially expressed genes were also detected in PSPC1gt knockout cells (Figure 1E), but a slight increase in upregulated gene expression was observed. Notably, Nono^gt^ and PSPC1^gt^ shared only approximately 40% of DEGs (Figure 1F).

Gene ontology (GO) analysis revealed that processes such as DNA replication and cell division were upregulated in Nono^gt^ at both time points, whereas genes related to cell adhesion, extracellular matrix organization, tissue development, and organ morphogenesis were similarly downregulated at CT6 and CT18 (Figure S3). Conversely, the main upregulated biological processes in PSPC1^gt^ were related to tissue embryonic development and cell differentiation, while inflammatory processes and immune responses, together with cell migration and adhesion, were downregulated (Figure S4), with no difference at the two time points.

These findings demonstrate the profound differences ability of Nono and PSPC1 to modulate distinct biological processes. Only NONO upregulates genes involved in DNA metabolism and cell cycle control, in accordance with the previously described phenotype.^1^

### Nono and PSPC1 modulate circadian transcription in phase phase-dependent manner

**Figure 2:**
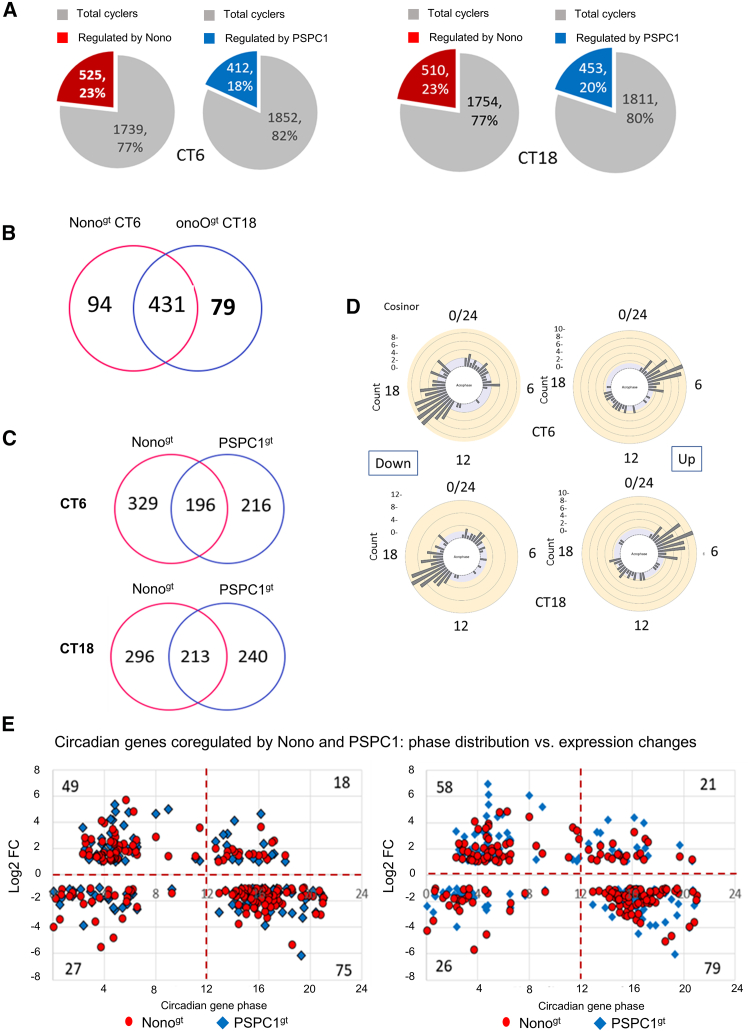
Regulation of the circadian transcriptome by Nono and PSPC1: unique and co-regulated aspects (A) Pie charts showing the number and percentage of circadian genes differentially regulated by Nono and PSPC1, at CT6 and CT18 (edgeR, FDR<0.01, |log2FC|>1). (B) Venn diagram shows the overlap of circadian genes regulated by Nono at CT6 and CT18 (Fisher’s exact test: *p*-value <2.2e-16, odds ratio 95.7). (C) Venn diagram displays the overlap of the circadian DEGs in Nono^gt^ and PSPC1^gt^ (Fisher’s exact test: CT6 *p*-value <2.2e-16, odds ratio 34.4; CT18 *p*-value <2.2e-16, odds ratio 37.3). (D) Rose diagrams show the phase distribution of the circadian genes that are commonly up- or down-regulated by Nono and PSPC1. (E) Plots show the distribution of the acrophase of circadian genes co-regulated by Nono and PSPC1 as a function of their differential expression.

Using the time points corresponding to the phase peaks of circadian genes in WT MEFs, we subsequently examined the impact of Nono and PSPC1 depletion on circadian gene expression. Interestingly, this analysis showed that both proteins play major roles in regulating the rhythmic expression of many genes. Indeed, on average, 20% of the total rhythmic transcripts were altered in Nono- and PSPC1-depleted cells (Figure 2A).

Moreover, the majority (80%) of the differentially expressed circadian genes in Nono^gt^ are common at both CT6 and CT18 (Figure 2B).

An overlap analysis also showed that Nono and PSPC1 share the regulation of the rhythmic expression of a marked number of genes. Indeed, 196 and 213 of differentially expressed circadian genes are common to both Nono and PSPC1 at CT6 and CT18, respectively (Figure 2C).

Considering the subset of circadian genes that are jointly up- and downregulated by Nono and PSPC1, (i.e., genes with differential expression in both depleted genotypes and with the same direction of expression change), we observed 169 and 184 genes at CT6 and CT18, respectively, with 70–75% overlap between the two time points. Interestingly, at both time points, the up-regulated genes exhibited phase peaks around CT4, which were almost opposite to the phase peaks of the down-regulated genes (Figure 2D).

This was confirmed by plotting the phase distribution of these coregulated genes versus their expression, which revealed a relationship with their circadian phase. Indeed, the upregulated circadian genes mainly peaked during the circadian daytime, whereas the downregulated ones mainly peaked during the circadian night. Surprisingly, the effect was similar at the two time points (Figure 2E).

### The transcriptional and post-transcriptional mechanisms of the Nono-mediated regulation of mRNA

#### Nono deficiency does not affect RNA nuclear retention

**Figure 3:**
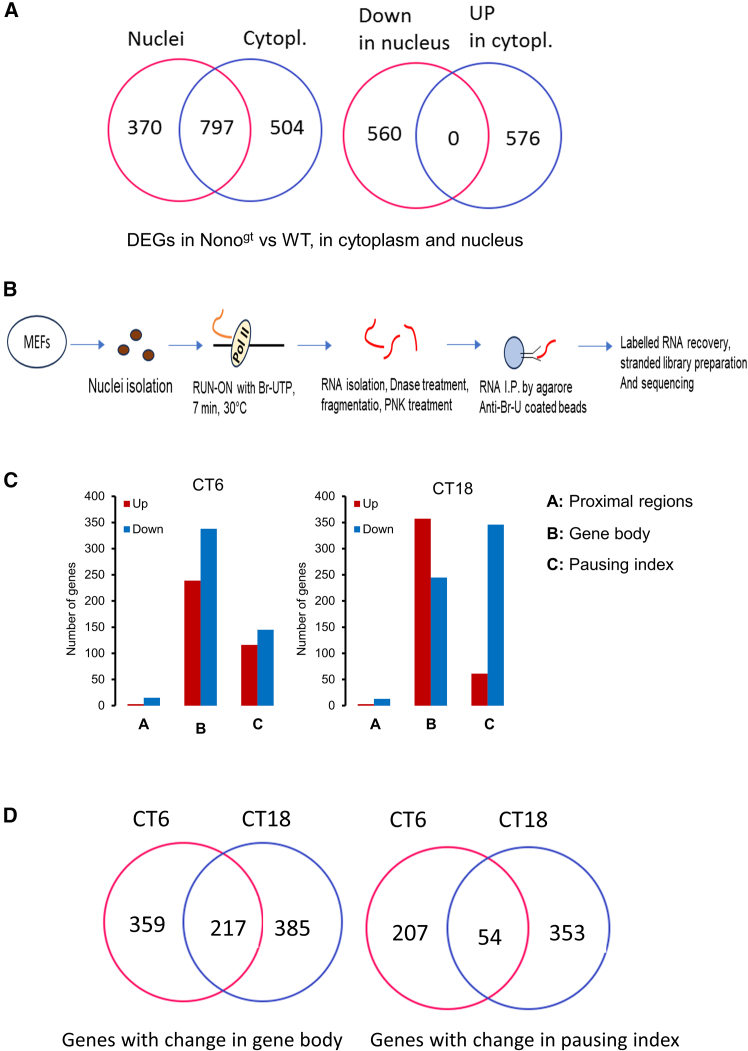
Effects of Nono upon transcriptional steps (A) Venn diagrams show the degree of DEGs in the nucleus and cytoplasm of Nono^gt^ compared to WT MEFs (Fisher’s exact test: DEGs in nuclei versus Cytoplasm *p*-value <2.2e-16, odds ratio 53.9; Downregulation in nuclei versus upregulation in cytoplasm: *p*-value = 1, odds ratio 0). (B) Schematic of the GRO-seq experiment. (C) Histogram summarizes the GRO-seq results. The graphs display the different number of active genes in Nono^gt^ versus WT MEFs at the indicated time points across the different steps of transcription. The analysis was performed by the Nascent RNA Sequencing Analysis (NRSA) tool (FDR<0.05). (D) Venn diagrams of the GRO-seq data, showing the overlap at CT6 and CT18 of the genes with differences in gene body (elongation) or pausing near the promoter (Fisher’s exact test: gene body *p*-value <2.2e-16, odds ratio 3.8; pausing index *p*-value 2.6e-5, odds ratio 2.0).

Nono is known to play a role in the retention of A-to-I hyper-edited RNA in nuclear paraspeckles.^32^ However, the presence and abundance of paraspeckles seem to depend on different kinds of triggers (chemical, mechanical, and so forth) Thus, in general, paraspeckles seem to mainly play a functional role when cells face external stressors.^33^ We decided to examine whether the observed changes in gene expression were partially the result of the altered retention of transcripts. To this purpose, nuclear and cytoplasmic mRNAs of WT and Nono^gt^ were separately analyzed, after the fractionation of the two cell compartments. The effectiveness of the fractionation is shown in Figure S5. This analysis revealed more than a thousand differentially expressed genes in both compartments, nucleus, and cytoplasm (Figure 3A**, left**). Moreover, the analysis showed that in NONO-deficient MEFs, 560 genes were markedly down-regulated in the nucleus and 576 genes were up-regulated in the cytoplasm, without any overlap (Figure 3A**, right**). This result indicates that in the two compartments, the genes are co-regulated and not anti-regulated and that, in our experimental conditions, the gene expression changes observed in NONO^gt^ are not the result of the retention of transcripts in the nucleus.

#### Nono controls both transcriptional elongation and the pausing index

Although Nono is devoid of a canonical DNA-binding domain, it has been suggested to act as a cofactor in transcription, with possible activities on initiation, elongation, and termination.^7^^,^^34^^,^^35^ To better define the critically regulated transcriptional steps by Nono, we performed a Global Run-On sequencing (Gro-Seq) experiment. This *in vitro* assay measures the short nascent transcripts associated with engaged polymerase, providing a precise map of polymerase density across active genes during initiation, elongation, and pausing (Figure 3B).^36^^,^^37^

Like RNA-seq experiments, Gro-seq was carried out at two time points after cell synchronization. This approach revealed a total of 4285 and 3364 genes to be transcriptionally active at CT6 and CT18, respectively.

In Nono-deficient cells, a very limited number of genes exhibited changes in transcription at the proximal promoter region, suggesting that Nono has little effect on the recruitment of polymerase and preinitiation complex formation. However, gene body transcription showed marked changes in Nono^gt^ cells, indicating a role for this protein in the regulation of the elongation. The comparison of the Pol II density in the paused peaks immediately downstream of TSS relative to the Pol II density in the body of the genes revealed strong changes in the pausing index, suggesting that the transition of Pol II from the paused to the productive elongation stage is an important step in the regulation of transcription by Nono (Figure 3C). Interesting differences in gene body transcription and in the pausing index between the two time points were also observed (Figure 3D).

As expected, a negative correlation between the transcriptional change in the pausing index and the change in the gene body region was observed, as well as a positive correlation between transcription at the promoter-proximal region and in the gene body (Figure S6A). These results demonstrated that Nono is directly involved in the regulation of transcription, mainly at the elongation and pausing steps.

#### Impact of Nono on post-transcriptional splicing

Coupling between transcription and alternative splicing is an important layer of gene expression regulation. Nono has been suggested to be involved in post-transcriptional processes such as alternative splicing.^38^ To better understand the role of Nono in splicing, we used JunctionSeq to calculate the differential exon usage, i.e., changes in the relative usage of exons (or part of exons) caused by the experimental condition.^39^ For an inner exon, a change in relative exon usage is usually due to the variation in the rate at which this exon is spliced into transcripts (alternative splicing). A validation of the differential exon usage is shown in Figure S6B.

**Figure 4:**
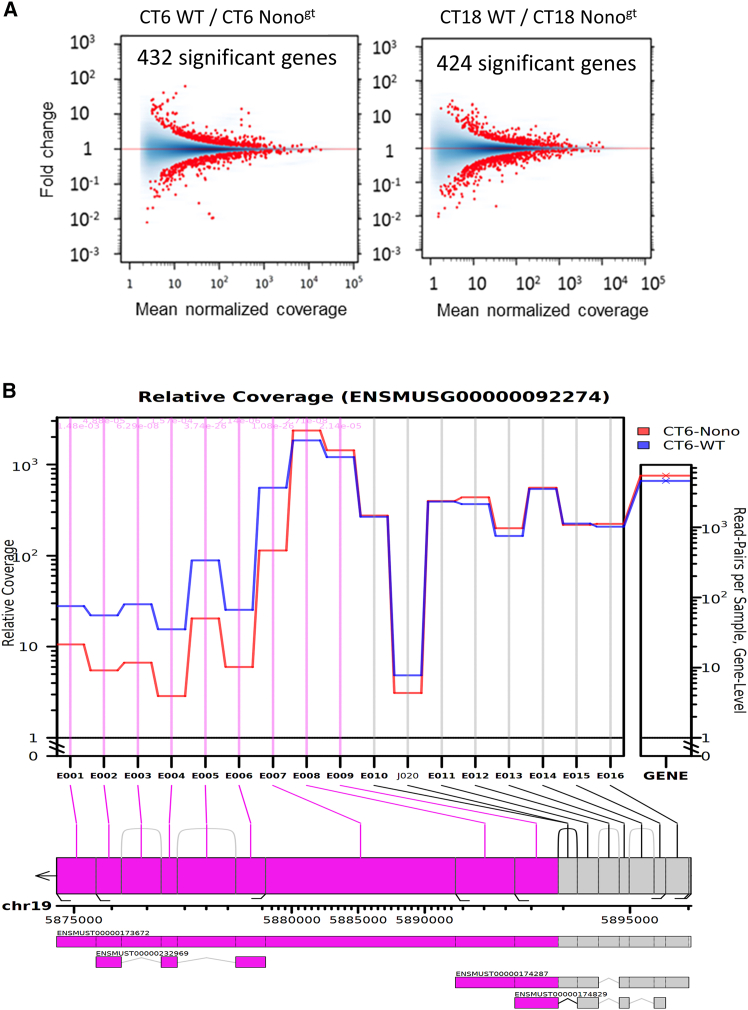
Nono regulation of post-transcriptional splicing (A) MA plots of log fold changes versus the mean of normalized counts showing the extent of differential usage of exons and splice junctions in Nono^gt^ versus WT at CT6 and CT18. The number in the plot shows the analysis at gene-level for the hypothesis that one or more features belonging to this gene are differentially used (JunctionSeq softwaare, GeneWise P adj. value <0.05). (B) JunctionSeq graph shows relative read coverage across the exon region of the *NEAT1* locus. The gene-wide expression is plotted in the right panel. The exonic regions being statistically significant (FDR<0.01) are marked with vertical pink lines. The annotated transcripts are displayed below the plot.

We observed that both Nono- and PSPC1-deficient cells exhibit a large change in splicing, much broader for PSPC1. Indeed, when compared Nono^gt^ to WT cells, 432 and 424 active genes showed differential usage of exon/junction at CT6 and CT18, respectively (Figure 4A). For PSPC1^gt^ the number of gene candidates was 4838 and 4586 at CT6 and CT18, respectively (Figure S7A). This broad effect of DBHS proteins on alternative splicing showed identical intensity at the two times of the circadian day.

However, the analysis also includes changes in the usage of alternative transcription start sites or polyadenylation sites, resulting in differential usage of exons at the 5′ and 3′ of transcripts. The latter is the case of the Neat1 gene: a single-exon gene originates two isoforms, sharing the same 5′-end but with alternative 3′-end processing.^40^ The result is a short isoform (Ensembl ENSMUST00000174287, Neat1-202, alias Neat1_1 in many publications) and a long one (Ensembl Transcript: ENSMUST00000173672, Neat1-201, alias Neat1_2).

As indicated in Figure 4B (CT6) the long and short isoforms (annotated below the graph) show that the regions just belonging to the long isoform (E001 to E007 in the graph) are under expressed in NONO^gt^, whereas the regions common to the two isoforms are overexpressed (E008-E009 in the graph). Because there is no different expression at the gene level, the result strongly indicates that the absence of NONO favors a switch from the long isoform to the short one. This finding is supported by the results in PSPC1^gt^: in fact, in these cells, the same Neat1 regions are under- and overexpressed as in Nono^gt^, with a global overexpression at the gene level, suggesting a common participation of the two DBHS proteins in Neat1 isoform switching (Figure S7B). The same effect was observed at both time points, either in Nono^gt^ and PSPC1^gt^.^40^ It is also noteworthy that the GO analysis of differentially spliced candidates in Nono^gt^ indicates the spliceosome assembly among the most enriched processes at both time points, suggesting a possible role of Nono in the regulation of the structural organization of the spliceosome itself. (Figure S8).

### Nono and R-loops

#### Nono regulates genome-wide R-loops homeostasis

Nono has been described as a multifunctional scaffold, acting with other proteins and/or RNAs.^5^ The observed effect of Nono on Pol II promoter-proximal pausing can be related to an effect on the DNA/RNA hybrids. Indeed, the protein has been shown to solve R-loops at telomeres,^21^ but whether Nono plays a general role in R-loops homeostasis is currently unknown. For this purpose, we decided to investigate if Nono affects the R-loop dynamics genome-wide.

We carried out detailed RNA/DNA immunoprecipitation (IP) analysis with the S9.6 antibody specific for RNA/DNA hybrids, followed by sequencing (DRIP-Seq) in WT and Nono^gt^ MEFs at the same time points as those used for RNA-Seq.^41^

Consensus peaks from triplicate samples were calculated for each condition relative to the input. The digestion of R-loops in the extracted DNA by RNase H before S9.6 immunoprecipitation was included as the negative control. A total of 46062 and 44996 peaks were obtained for CT6 and CT18, respectively. The specificity of R-loops detection by S9.6 antibody, as well as examples of the distribution of DRIP-seq peaks, are shown in Figures S9A–S9C.

**Figure 5:**
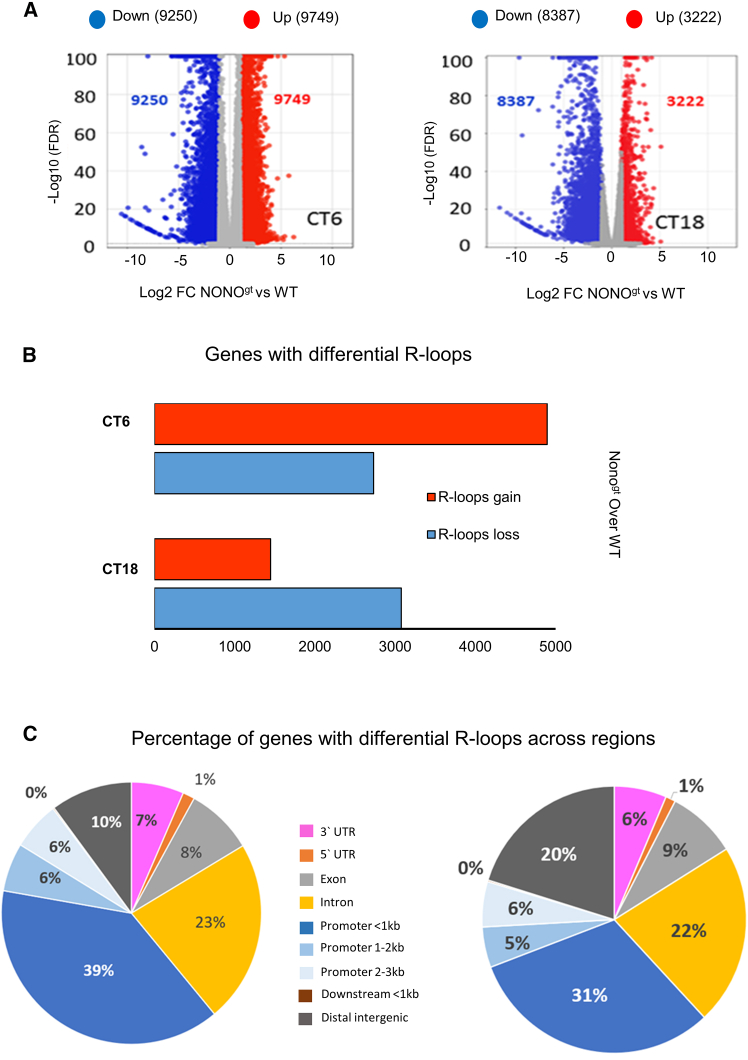
Nono protein regulates non-classical DNA structure (A) Volcano plot shows the differential presence of R-loops in Nono^gt^ versus WT at CT6 and CT18 (EdgeR, FDR<0.01, |log2FC|>1). (B) Bar graph shows the number of genes in Nono^gt^ vs. WT with significant increase or decrease of R-loop peaks intensity, termed R-loops gain and loss respectively, at CT6 and CT18 (EdgeR, FDR<0.01, |log2FC|>1). (C) Pie charts show the percentage of genes with differential R-loop expression across their regions, at the indicated time points.

We then analyzed the impact of Nono depletion on the DNA/RNA hybrid structure. Surprisingly, in Nono-deficient cells, we observed a dramatic genome-wide change in the R-loops landscape. We found a very high number of differentially expressed R-loop peaks in Nono^gt^ versus WT cells. More than 9000 peaks had either increased (defined R-loops gain) or reduced signal intensity (defined R-loops loss) in Nono^gt^ (DexSeq, FDR<0.01, log2FC > 1). Interestingly, a marked difference in the total number of differentially expressed R-loop peaks was observed with circadian time (CT6 vs. CT18), as well as in the ratio of R-loops gain and R-loops loss (Figure 5A). The intergenic R-loop peaks and peaks observed in genes that did not reach the threshold of expression in the RNA-seq (untranslated genes) were excluded from the subsequent gene analysis (Tables S2 and S3;Mendeley Data: http://www.doi.org/10.17632/63rjyv54w4.2).^42^

The number of genes expressing differential R-loops was very different at the two time points. Indeed, the number of genes with R-loops gain was much greater (4896) than that with R-loops loss (2730) at CT6. In contrast, at CT18, more than double the genes had R-loops loss (3076) than gain (1448), suggesting circadian dynamics in R-loops (Figure 5B).

A direct comparison indicates that the R-loops are not merely a by-product of variations in gene expression (Figures S10A and S10B).^43^

Altogether, our results show that although R-loop changes due to variation in transcription are present as expected, they do not account for the deep R-loop gain and loss in Nono^gt^. The results instead favor a genome-wide regulatory role for Nono on R-loop dynamics.

#### R-loops gain and loss in the promoter of circadian genes is time dependent

The effect of R-loops depends on their position within the gene, and the existence of R-loops classes based on their position and functional significance has been proposed.^44^ Thus, we determined the gene region distribution of the differentially expressed R-loops at the two time points. The highest percentage of genes had R-loop variation in their promoter (51% and 42% of genes at CT6 and CT18, respectively) versus 31% of the genes with R-loops variation in the gene body and 7% of the genes with R-loops changes at the 3′UTR at both time points (Figure 5C). Although this pattern was similar at the two time points, the number of genes with differentially regulated R-loops in the promoter was much lower at CT18 than at CT6.

The R-loops in the promoter regions have been suggested to favor or inhibit gene transcription.^45^^,^^46^ Considering the genes with R-loops in the promoter, we observed significant overlap between the DEGs and the genes with R-loops variation in their promoters, regardless of the positive or negative direction of the two variables, (Fisher’s exact test: CT6 odds ratio 1.40, *p*-value 1.1e-7; CT18 odds ratio 2.90, *p*-value 2.2e-16) (Figure S11A). The overlap was more significant when we focused on the circadian genes (Fisher’s exact test Odds ratios 17.63 and 25.65 at CT6 and CT18, respectively) (Figure S11B).

**Figure 6:**
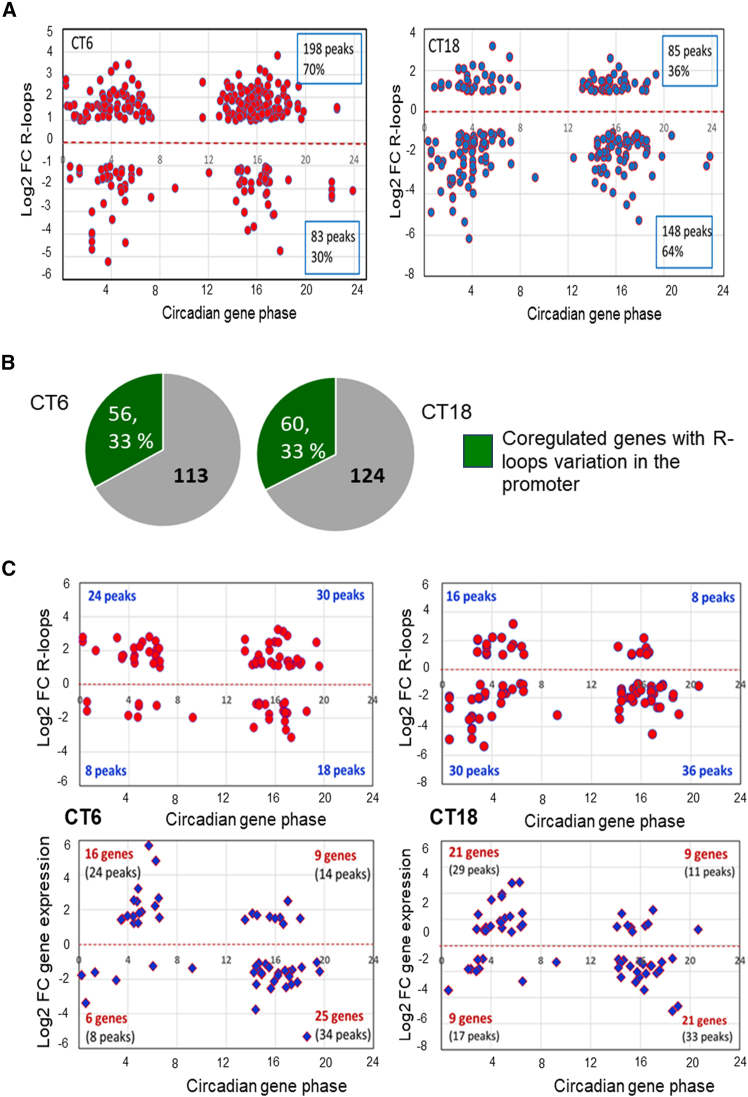
Relationship between R-loops in the promoter regions and gene expression in Nono-regulated genes (A) Scatterplots show the distribution of the R-loops fold changes in the promoter regions of the circadian genes regulated by Nono as a function of gene acrophase. (B) Pie charts show the circadian genes co-regulated by Nono and PSPC1. In green, the number and percentage of genes with R-loops variation in their promoter region at the two time points. (C) Upper panel: scatterplots show the distribution of the R-loops fold change in the promoter of the circadian genes co-regulated by Nono and PSPC1 as a function of gene acrophase. Note the time dependency. Lower panel: scatterplots of the distribution of the fold change expression of the corresponding genes versus gene phase. Note that gene expression indicates phase dependency at both time points.

However, by plotting the phase of these circadian genes versus the log2 FC of R-loops expression, we observed that the distribution was strongly time-dependent, with the preferential upregulation of R-loops at CT6 (70%) and downregulation at CT18 (64%) (Figure 6A).

Thus, despite the high overlap of circadian gene expression in Nono^gt^ at these two time points (more than 80%, Figure 2B), the R-loop regulation by Nono appears very different between CT6 and CT18. These findings further indicate that the R-loop gain and loss at circadian genes are not a consequence of gene expression changes. Alternatively, R-loops seem to follow a circadian presence, and they may have opposite effects on promoting gene expression, in agreement with the bimodal effect of the R-loop.^15^

#### Rhythmic R-loops can explain the phase-dependency of co-regulated genes

As described above, the Nono/PSPC1 complex co-regulates the circadian genes in an unexplained phase-dependent manner (Figures 2D and 2E). Although the DRIP experiment was carried out only for Nono, we hypothesized that co-regulation between Nono and PSPC1 may also occur for R-loops. We then investigated the relationship between the expression of the co-regulated genes and the variation in R-loops in their promoters in Nono^gt^.

One-third of the co-regulated genes had differentially expressed R-loops in the promoter, with 50% overlap at the two time points (Figure 6B). By plotting the R-loops gain and loss in the promoter versus the acrophase of the corresponding genes at CT6 and CT18, we observed the time-dependent regulation of R-loops, mainly the upregulation of R-loops at CT6 and downregulation at CT18, independent of the gene phase. On the other hand, by plotting the acrophase of the same genes versus gene expression, the picture changed completely: the up- or downregulation of gene expression appeared phase-dependent (more downregulated genes in the second part of the day and more upregulated genes in the first part), and the behavior was the same at the two time points (Figure 6C). A bimodal opposite effect of the promoter R-loops can potentially determine the observed phase dependency of co-regulated circadian genes.

### Nono controls the extent of DNA damage through R-loops

Our results above demonstrated a role for Nono protein in R-loop homeostasis. Given the deleterious effect of the accumulation of these genomic structures at DNA damage sites, we examined the role of Nono in a cell model involving replication stress, R-loops accumulation, and DNA damage.

**Figure 7:**
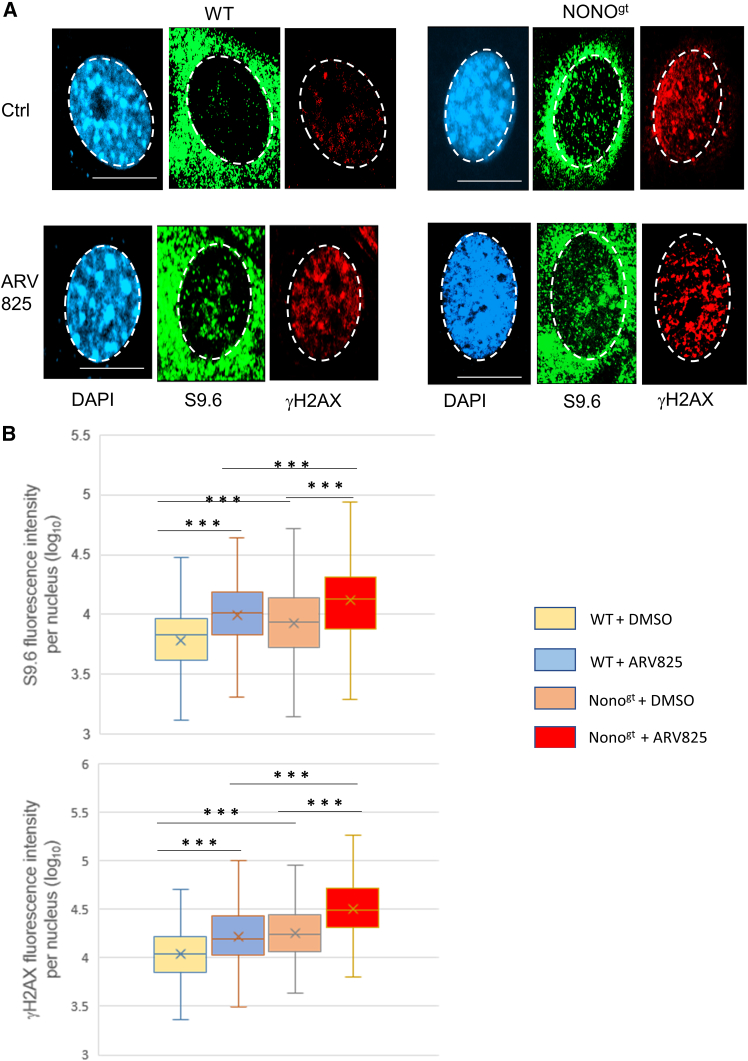
Nono prevents the R-loop accumulation and the associated DNA damage (A) Representative images of nuclear S9.6 (anti-mouse Alexa Fluor 488) and γH2A (anti-rabbit Alexa Fluor 594) immunofluorescence intensity in WT and NONO^gt^ cells after treatment with ARV-825 30nM or DMSO for 6 h. DAPI was used for counterstaining. Scale bar 15 μM. (B) Boxplots show the analysis of S9.6 and γH2AX integrated fluorescence per nucleus (300 nuclei analyzed per condition). The data were log transformed to obtain normal distribution (Shapiro-Wilk test, significance level *p* > 0.05) and equal variances (Levene’s test, significance level *p* > 0.05). two-Way ANOVA test showed significant differences of the means for both factors (genotype and treatment, significance level *p* < 0.05), without interactions (*p* = 0.55 for interaction). The result of Tukey HSD post-hoc test for multiple comparison of the means of different groups is indicated (∗∗∗ <0.001).

We used the BRD4 inhibitor ARV-825, which has been shown to cause both DNA damage and R-loop accumulation.^47^ WT and Nono^gt^ MEFs were first treated with AV-825 30 nM or vehicle for 6 h, then cells were fixed and stained with antibodies S9.6 (specific for R-loops) and γH2AX (a marker of DNA damage). We first noticed that in Nono^gt^ control cells, there was a spontaneous higher R-loop level compared to control WT. The same comparison of the two genotype controls also evidenced higher DNA damage in Nono^gt^, indicating higher levels of replication stress under basal conditions in Nono-depleted cells (Figures 7A and 7B). Consistently, a significant increase in DNA damage (γH2AX signal) was also observed in cells treated with ARV-825. However, this increase is markedly higher in Nono-deficient cells. Interestingly, in BRD4-inhibited cells, we also observed a parallel increase of R-loops and of S9.6 fluorescence signal, significantly higher in Nono^gt^ MEFs (Figures 7A and 7B). We have also shown a reduction of S9.6 fluorescence intensity per nucleus when RNase H1-EGP was transiently expressed in WT MEFs and a concomitant significantly lower level of γH2AX signal (Figures S12A and S12B).

Altogether, these results confirm a causal relationship between R-loop accumulation and DNA damage after BRD4 inhibition. They also support a role of Nono upon DNA damage through R-loop modulation.

## Discussion

### Circadian and non-circadian aspects of the Nono regulation of transcriptome

Nono has been described at a single gene level as either a coactivator or corepressor. To investigate this further, we examined whether Nono could modulate expression genome-wide, playing a coactivator and corepressor role at different times of the circadian cycle. Transcriptome analysis in Nono-deficient MEFs demonstrated that Nono can both increase and decrease gene expression, but with highly similar effects at the two time points. These findings indicate that Nono does not switch between activator and repressor functions across the circadian cycle (Figure 1D). A similar pattern was also shown by PSPC1 (Figure 1E).

A circadian aspect in transcriptome regulation was particularly evident when looking at circadian genes. Among the obligatory homo- or heterodimers formed by Nono and PSPC1 only the NONO/PSPC1 complex has a circadian role, regulating a common set of circadian genes in a phase-dependent manner (Figure 2). The identical effect observed at CT6 and CT18 rules out a phase shift or loss of oscillation in NONO/PSPC1-deficient cells as an explanation for this surprising behavior.

The pre-mRNA alternative splicing regulation by Nono and PSPC1 did not show circadian time dependency: the effects at the two time points are identical, either for Nono or PSPC1. If the complex Nono/PSPC1 has a circadian role, this result suggests that alternative splicing may be regulated independently by the two DBHS proteins. This hypothesis is further supported by the different magnitude of the effect on exon usage. In fact, after confirming the engagement of Nono in the pre-mRNA splicing (as previously described^38^ (Figure 4A), we analyzed the role of PSPC1. PSPC1 is a well-known component of paraspeckles. However, regarding other roles of PSPC1, there are only limited studies, and its functions are not completely understood. Surprisingly, our analysis revealed that PSPC1 has a higher impact on differential exon usage than Nono (Figure S7A). This finding warrants further investigation and confirmation.

We further observed a potentially important effect on the switching between long and short isoforms of Neat1 either in Nono and PSPC1-deficient cells (Figures 4B and S7B). DBHS proteins are major components of paraspeckles, and in turn, the formation of paraspeckles depends on the transcriptional readthrough of Neat1_1 to Neat1_2; therefore, an effect on the switching between the two isoforms is of great importance. DBHS proteins appear to promote the switch to the long isoform, possibly facilitating paraspeckles formation. Notably, the Neat1 isoform switching has been demonstrated to play a role in some cancers, although the oncogenic relevance of the two isoforms needs to be further clarified.^40^

A time-dependent effect of Nono was revealed by the dissection of the transcriptional steps. Nono has been previously demonstrated to interact with the carboxyl-terminal domain of the RNA polymerase II, and it was suggested to possibly favor the binding of RNA and pol II.^48^ However, our findings indicate greater effects of Nono on promoter-proximal pausing and elongation. These two transcriptional steps have been previously shown to be related.^49^ Interestingly, the number of genes affected by Nono at these steps is largely different at the two time points, especially for promoter-proximal pausing (Figure 3F).

### R-loops, NONO, and circadian gene expression

The identification of Nono in the R-loop interactome and its effect on Pol II pausing, a process linked to R-loops, led us to investigate these DNA/RNA hybrid structures. Genome-wide mapping of R-loops in Nono-deficient and wild-type (WT) MEFs revealed a greater effect on R-loop distribution across the genome than expected. This represents the first demonstration of the importance of Nono in R-loop homeostasis.

Many R-loop readers and regulators have been described, and new ones are continuously found. Besides the Ribonuclease Rnase H that resolves R-loops and Topoisomerases that prevent their formation, the main categories of regulators include, for example, the helicase family, the DNA Damage Repair factors, and some transcription and RNA processing factors. Compared to most regulators, the Nono modulation of R-loop homeostasis shows unique features. In fact, the different kind of regulators mainly plays a selective role, favoring the resolution or the formation of R-loops. The helicases are a good example: they possess either strand-annealing activity that promotes the hybridization of RNA to DNA and an unwinding activity that separates RNA–DNA hybrids and resolves R-loops. The Ddx19 (member of the DEAD-box family), upon DNA damage and activation of the ATR/Chk1, relocates to the site of DNA damage to promote R-loops resolution.^50^ On the contrary, the helicase DHX9 favors the formation of R-loops: if the RNA splicing is defective, it associates with the Pol II, inducing R-loops formation and the trapping of Pol II to the chromatin.^51^ Senataxin is another helicase which associates with BRCA1 (a DNA Damage Repair factor) at the sites of PolII pausing at transcription termination sites to promote R-loops resolution.^52^^,^^53^ In the case of Nono, it appears to be able to determine either an increase or a decrease of R-loops (Figures 5A and 5B). When a simultaneous gain and loss of R-loops is reported, like in DDX1 knock-out cells, the magnitude of the changes (638 increased R-loop regions and 1058 decreased R-loop regions) is much lower than that observed in Nono^gt^ (more than 9000 R-loops gain and loss at CT6, 8000 R-loops gain and 3000 R-loops loss at CT18) (Figures 5A and 5B).^54^ In another DRIP study of the R-loops regulation by the three factors DDX5 (a DEAD-box helicase), XRN2 (5′-3′ exoribonuclease that associates with Senataxin), and PRMT5 (a protein arginine methylase), the authors observed the expected R-loops increase, with a number of peaks gain ranging from 700 to 2000 in the knock-out cells.^55^ On the whole, Nono appears quite unique in its ability to regulate the R-loops distribution in a vast number of genes. This feature of Nono suggests the involvement of several mechanisms in its regulation of R-loop homeostasis.

A very interesting topic is the relationship between circadian gene expression, the molecular clock, and R-loops.

More than 30% of R-loop peaks were found in the promoters, and in NONO-deficient cells, a marked number of genes showed a variation of R-loops in their promoter regions, with both an increase and decrease of R-loop peaks compared to WT. Promoters are a hotspot for R-loop formation, which strongly suggests their regulatory role in gene expression.^15^ The level of a gene expression depends on chromatin architecture and the accessibility of the transcription machinery to DNA. In this regard, R-loops are more and more identified as regulators of the chromatin state. For example, the promoter regions with the asymmetric distribution of cytosine guanine residues, called CG skew, are particularly prone to R-loops formation: in these regions there is a generally negative correlation between R-loops and DNA methylation and a positive association with marks of active transcription such as H3K4me3.^56^^,^^57^ However, R-loops at promoters can also repress gene expression. An example is the silencing of the FMR1 gene in the Fragile X syndrome by R-loops formed by FMR1 RNA and FMR1 DNA: in this case, the R-loops are associated with histone repressive marks. Therefore, sometimes R-loops can determine chromatin condensation, in other cases open chromatin, for unclear reasons. The effect seems to be gene-specific.^57^

The molecular clock can influence chromatin accessibility in circadian genes and, consequently, their circadian expressions, as recently investigated in Drosophila.^58^ The authors found a circadian oscillation of chromatin accessibility at dusk and dawn, related to the peaks of expression of the circadian genes. They propose that the molecular clock is responsible for a circadian variation of chromatin accessibility, therefore determining the rhythmic oscillation of these genes.

To the best of our knowledge, the link between the circadian clock machinery and the R-loop regulation has not yet been investigated in mammals. However, our findings reveal time-dependent variations in R-loops at the promoters of Nono-regulated circadian genes (Figure 6A). This provides the first evidence of circadian dynamics in R-loop regulation and of the connection between the molecular clock and the presence of R-loops in the promoter of circadian genes.

The link between the circadian clock and R-loops in the promoters can be important in regulating the chromatin accessibility and the expression of circadian genes. The temporal regulation of the presence of positive and negative transcriptional regulatory R-loops during the circadian day could affect both rhythm and phase of gene expression. Specifically, considering the Nono/PSPC1 co-regulated genes, we propose that two subsets exist. The first subset of genes (peaking in the first part of the circadian day) can possibly have positive transcriptional R-loops in their promoters in the first part of the day and negative R-loops in the second part. This means that the accessible chromatin and its peak phase are in the first part of the day, while the lower expression in the second part is due to a circadian shift of the chromatin state. Nono absence induces R-loop gain in the promoter in the first part of the day (Figure 6A), which can lead to an increased gene expression. Equally, a loss of negative transcription R-loops in the second part of the day results again in a higher gene expression (Figure 6A). For the other subset of genes, which have the phase peak in the second part of the day, the opposite presence of negative and positive transcriptional R-loops in the first and second part of the circadian day can be linked to opposite variations of the chromatin state. In this case, an increase of negative transcription R-loops, as observed at CT6, and a decrease of the positive ones at CT18 (Figure 6A) result in a similar decrease of expression. This is what we observed in Figures 6B and Figure 2E: a phase-dependent regulation of the Nono/PSPC1 co-regulated circadian genes, identical at the two time points. This result agrees with a possible temporal modulation of the R-loops by the circadian clock.

An important question can be stated of how the circadian clock regulates the chromatin accessibility and R-loops, and how Nono can affect it. Prior research demonstrated an essential role of the core clock protein Per in the circadian oscillation of the chromatin state in Drosophila.^58^ Nono links the circadian clock and the cell cycle by modulating the Per activity in mice.^1^ We can only hypothesize a similar mechanism in the case of the connection Clock/R-loops. It would be very interesting to examine how much the Per1/Per2 null condition recapitulates the R-loop changes observed in Nono-deficient cells.

### NONO and DNA damage: R-loops involvement

DNA-RNA hybrids have been shown to accumulate at the site of DNA damage and must be timely removed to avoid replication stress, genome instability, and DNA breaks. At the same time, their presence can contribute to DNA repair by inducing the relocation of factors involved in DNA Damage Repair (DDR).^15^^,^^16^ Therefore, it is important to understand how the R-loops deregulation in the absence of NONO affects DNA damage.

The participation of Nono in DDR has been suggested to rely on different mechanisms. For example, Nono has been shown to bind TOPBP1 and favors its chromatin loading to activate the fundamental DNA damage and replication checkpoint ATR-Chk1.^59^^,^^60^ In this study we used the BRD4 inhibitor ARV-825 as a damaging agent because BRD4 is required for the activation of ATR-ChK1 checkpoint through the topoisomerase II-binding protein 1 (TOPBP1) and its inhibition is known to alter elongation, RNAPII stalling, further determining R-loops accumulation, collisions with the replication machinery and DNA breaks.^47^ In this cell model, the absence of Nono makes the DNA repair system defective and unable to properly limit the unscheduled R-loops. The presence of Nono can clear the R-loops, possibly via the activity of DDX19 activated by ATR.^48^

A complex SFPQ/Nono has been shown to be present at the sites of DNA damage and modulate the radioresistance in human cells. Sequences in the SFPQ protein seem to be important in mediating this localization, and the complex can determine DNA-PK activation by autophosphorylation.^61^ This is not in contradiction with our result, as it is possible that Nono is present at the DNA damage sites in a complex with SFPQ and that it affects more than one pathway (DNA-PK and ATR). Although the SFPQ/Nono and the ligase complex XLF/XRCC4 work in the common NHEJ pathway, the same Dynan lab showed that the Nono subunit of the DBHS complex has additional functions for DDR in damage induced by radiation or cisplatin.^62^ The authors speculate that the RNA-binding capacity of Nono can be responsible for these functions. One possibility suggested by the authors is that the RNA binds to lncRNAs generated at the site of DNA damage. However, these lncRNAs can be one of the sources of R-loops.^15^ This would confirm the R-loops as important mediators of the Nono activity in DDR.

A second suggested possibility is that the RNA binding of Nono modulates other genes involved in DNA Damage Repair. This link between DDR and RNA metabolism, mediated by Nono, has also been recently suggested by another group. In cancer cells, the treatment with etoposide or bleomycin induces the increased expression of the DDR factor growth arrest and DNA damage-inducible beta (Gadd45b), which is reduced in Nono-deficient cells. Because of the known binding of Gadd45a to R-loops, the authors speculate that Gadd45b can also be recruited to R-loops at DNA damage sites and can work together with Nono to modulate RNA Pol II and resolve R-loops.^63^ Gadd45a and Gadd45b are highly homologous (they can also form heterodimers) and share the ability to recruit nucleotide and/or base excision repair factors to gene-specific loci. The increased expression of Gadd45b by Nono in the case of DNA damage is then a further way to promote genomic stability.

The Etoposide treatment also has a more general effect on Nono and RNA metabolism, involving another type of R-loops.^64^ Following exposure of human cancer cells to this agent, the authors observed a synthesis by Pol II of non-coding antisense transcripts from nucleolar spacers. These transcripts form R-loops and determine the relocation of NONO to the nucleolus. The expression of highly expressed NONO-dependent genes is reduced, with the consequent possible formation of R-loops, while NONO retains some pre-mRNA transcripts in the nucleolus, favoring DDR.

The dependency on R-loops for the presence of Nono at DNA damage sites has been previously demonstrated. Poly (ADP-ribose) polymerase 1 (PARP1) is recruited and activated at the sites of double-strand breaks (DBS) by binding to R-loops and promoting their resolution.^65^ In turn, the Nono recruitment to DNA damage sites is dependent on poly (ADP)-ribose (PAR), generated by the activated PARP-1.^66^

However, our results contrast with those reported by Li S. et al.^67^ In Nono knockout mouse embryonic fibroblasts, the authors found an increased expression of PSPC1, which compensates for Nono deficiency. The results of the current study show that the roles of DBHS proteins function in DNA repair are not interchangeable. Several possibilities might explain this discrepancy, such as a difference in the mouse genetic background or a difference in the penetrance of the phenotype. For example, Li S. et al. found a similar growth rate between WT and Nono knockout MEFs, while we observed a higher proliferation rate in NONO^gt^, consistent with previous findings from our lab.^1^ A higher proliferation rate can provoke replication stress that can confer more sensitivity to further DNA damage. Moreover, the difference in the DNA-inducing agent (γ-rays versus BRD4 inhibition) can provide an additional explanation of the different results. In this respect, a recent study showed that Nono is the only DBHS protein degraded after UV-induced DNA damage, to turn off the ATR-CK1 checkpoint, suggesting a stimuli-dependent function of the DBHS proteins.^24^

Overall, the ways Nono interacts with the sites of DNA damage and the activities it plays to prevent genomic instability can be various, most of them involving the DNA-RNA hybrid formation.

Our investigation of the role of Nono in DNA damage was carried on in cells without molecular clock synchronization. However, significant links between the circadian clock and genomic instability have been reported by many studies. The complex interplay is deeply discussed in an excellent review, with its relevance for cancer.^68^ In the presence of DNA damage, the DDR shows a rhythmicity dictated by the circadian clock and the core clock genes generally favor the DDR. Alterations of the pathways that connect the clock and the DNA damage impair the DNA repair, resulting in genomic instability and possibly cancer development. For example, after UV-induced DNA damage in fibroblasts, the ATR-Chk1 signaling pathway is activated to allow nucleotide excision repair (NER), and this pathway shows oscillation in a 24-h period. This rhythm is dependent on the core clock genes CRY1 and Timeless (TIM).^69^

In the context of DNA damage, a tumor suppressive function has been suggested for the three PER genes (PER1, PER2, PER3). In fact, a DSB activates the ATM-Cdk2 kinase and the DSB pathways, activates p53, and induces apoptosis in cancer cells. All three PER genes play a role in Cdk2 activation and promote apoptosis (PER1 is specifically described in).^23^

The core clock gene Timeless has an important role in DDR regulation. It acts as an accelerator of DNA fork replication. In case of DNA damage upon the production of reactive oxygen species (ROS), it works in a complex as a redox sensor. This causes its dissociation from the replisome and slows down the fork progression to reduce replication stress.^68^ In our RNAseq analysis, we observed that Timeless is significantly overexpressed in Nono-deficient cells. If this result is confirmed in RNA-seq after DNA damage, Timeless can serve as a link between Nono and genome stability.

Furthermore, the clock regulation of cell cycle checkpoints is potentially relevant for Nono. Bmal1 regulates the circadian expression of p21, hence the transition from G1 to S phase. P21 has a function in genomic stability by inducing cell-cycle arrest after DNA damage, and its dysregulation can determine replication stress and genomic instability. Nono also regulates the G1 to S phase transition through a circadian and PER-dependent modulation of p16Ink4 expression,^1^ protecting against replication stress. In fact, in our DNA damage model, Nono^gt^ cells showed higher basal levels of γH2AX compared to WT.

The participation of R-loops in the circadian clock control of DDR is a very compelling research topic. However, until now, this relationship has not been investigated. We are aware only of preliminary data in Arabidopsis thaliana.^70^ In this plant, the molecular clock regulates the circadian rhythm of DDR response to bleomycin damage. The main player for this regulation seems to be the core clock gene CRY2, which induces the increased expression of genes relevant to DDR. CRY2 plays its function in DDR regulation in Arabidopsis thaliana through changes in R-loop formation and subsequent modification of the chromatin state.

### Conclusion and outlooks

Our data provide a detailed understanding of how much Nono influences transcription and RNA splicing genome-wide. We then highlighted its key role in regulating R-loop homeostasis and dynamics.

It will be very interesting to identify the Nono domains necessary for R-loops reading. In fact, intrinsically disordered protein regions (IDR), present in DBHS proteins, have been proposed to mediate the direct interaction with R-loops, inducing liquid-liquid phase separation.^71^ In this regard, the DNA damage-induced paraspeckles have been proved to enhance the DDR through the autophosphorylation of DNA-PK.^23^^,^^72^

How much the R-loops modulation can be involved in the multiple effects of Nono on RNA metabolism is a further interesting topic.

Understanding DBHS proteins’ interactions with DNA and RNA structures, such as R-loops, will not only advance our knowledge about their role in the regulation of transcriptional and post-transcriptional processes but could also provide a new therapeutic target, especially against cancer.

### Limitations of the study

SFPQ was not included in the study because mice homozygous for a null allele die embryonically, and it was not possible to obtain homozygous MEFs. Moreover, the exact molecular mechanism by which NONO regulates R-loop dynamics is still lacking. To address this, one could explore the potential NONO interacting partners known to be involved in R-loop regulation. Furthermore, the potential role of other DBHS proteins in R-loop dynamics is still lacking, opening possibilities for further research.

In summary, our work showed that the circadian clock is responsible for the daily variations of R-loops, particularly at circadian gene promoters. Therefore, further studies are needed to address the exact contribution of individual core clock genes to these dynamics.

## Resource availability

### Lead contact

Further information and any requests should be directed to and will be fulfilled by the lead contact, Melissa Pisteljic (melissa.pisteljic@uzh.ch).

### Materials availability

This study did not generate new unique reagents.

### Data and code availability

• Data: Raw data reported in this article have been deposited at the European Nucleotide Archive (ENA) accessible on https://www.ebi.ac.uk/ena/browser/view/PRJEB76991 and at Mendeley Data accessible on https://data.mendeley.com/datasets/63rjyv54w4/2^42^ and are publicly available as of the date of publication.
• Code: This article does not report original code.
• Any additional information required to reanalyze the data reported in this article is available from the lead contact upon request.

## Acknowledgments

This study was funded by the grant #179313 (DBHS family proteins in clocks and sleep: nuclear and synaptic mechanisms) from the 10.13039/501100001711Swiss National Science Foundation (SNSF).

A. A is supported by the grant # MAB/2017/2 (Regenerative Mechanisms for Health - ReMedy project).

The authors thank the Functional Genomic Center Zurich for the accurate sequencing and data analysis.

We also thank all members of S.A. Brown lab for the deep discussion of the results and the suggestions during the project.

## Author contributions

S.A.B. and E.M. conceived and designed the experiments, S.A.B. received funding. E.M. performed experiments. L.O., E.M. and A. R-A. performed data analysis. E.M., A.A. and M.P. wrote the article. A.A. and M.P. provided expertise and feedback.

## Declaration of interests

The authors have no conflicts of interest to declare.

All co-authors have seen and agree with the contents of the article and there is no financial interest to report. We certify that the submission is original work and is not under review at any other journal.

## STAR★Methods

### Key resources table

**Table:** 

REAGENT or RESOURCE	SOURCE	IDENTIFIER
**Antibodies**		

BrdU Antibody (IIB5) Agarose Beads conjugated	Santa Cruz Biotechnologies (USA)	Cat# sc-32323 AC; RRID:AB_626766
Anti-DNA_RNA Hybrid, clone S9.6, from mouse	Millipore (USA)	Cat# MABE1095; RRID:AB_2861387
pAB to gamma-H2AX (phospho S139), from rabbit	Abcam (USA)	Cat# 11174; RRID:AB_297813
Goat, secondary antibody, Alexa Fluor 594 anti-rabbit	Thermo Fisher Scientific (USA)	Cat# A66787; RRID:AB_3251387
Goat, secondary antibody, Alexa Fluor 488 anti-mouse	Thermo Fisher Scientific (USA)	Cat# A-10680, RRID:AB_2534062

**Chemicals, peptides, and recombinant proteins**		

Puromycin	Sigma-Aldrich (DE)	Cat# P4512
D-Luciferin free acid	Regis Technologies (USA	Cat# 1G
RNase-OUT	Thermo Fisher Scientific (CH)	Cat# 10777019
SUPERase In RNase Inhibitor	ThermoFisher Scientific (CH)	Cat# AM2696
5-Br-UTP	Jena Bioscience (DE)	Cat# NU-121
RNA fragmentation reagents	Invitrogen (UK)	Cat# AM8740
Micro Bio-Spin P-30 Gel Columns	Bio-Rad (CH)	Cat# 7326250
T4 Polynucleotide Kinase (PNK)	ThermoFisher Scientific (CH)	Cat# EK0032
Proteinase K	Thermo Fisher Scientific (CH)	Cat# EO0491
high-density Maxtract phase-lock gel tube	Qiagen (D)	Cat# 129065
phenol/chloroform/isoamyl alcohol (25:24:1).	Roth (CH)	Cat# A156.3
BsrGI	New England Biolabs (USA)	Cat# R0575S
EcoRI	New England Biolabs (USA)	Cat# R0101S
HindIII	New England Biolabs (USA)	Cat# R0104S
SspI	New England Biolabs (USA)	Cat# R0132S
XbaI	New England Biolabs (USA)	Cat# R0145S
NEB CUT SMART Buffer	New England Biolabs (USA)	Cat# B7204
Spermidin Trihydrochlorid	Thermo Fisher Scientific (CH)	Cat# 10300904
2-mL phase-lock gel, light	5PRIME (DE)	Cat# 733-2477
RNase H, recombinant	New England Biolabs (USA)	Cat# M0297S
Protein A-Agarose	Roche Diagnostics GmbH (DE)	Cat# 11719408001
12 Well Chamber	Ibidi (D)	Cat# 81201
Fibronectin	Sigma-Aldrich (D)	Cat# F1056
DAPI	Sigma-Aldrich (D)	Cat# 9542
Dako	Agilent Technologies AG (CH)	Cat# HY-16950
Normal Goat Serum	Biorad (D)	Cat# C076A
ARV-825	Sigma-Aldrich (D)	Cat# SML3423
TransIT-2000 transfection reagent	Mirus Bio (USA)	Cat# MIR5404
Opti-MEM	GIBCO-Thermo Fisher Scientific (CH)	Cat# 11058-021
12 Well Chamber	Ibidi (D)	Cat# 81201

**Critical commercial assays**		

Mycoalert detection kit	Lonza (USA)	Cat# LZ-LT07-218
Total RNA Purification Micro Kit	Bio-Tek (CAN)	Cat# 35300
RNeasy MIDI Kit	Qiagen (DE)	Cat# 75142
Illumina TruSeq mRNA Library Prep	Illumina (USA)	Cat# 20020594
Illumina TruSeq Total RNA Library Prep	Illumina (USA)	Cat# 20020596
Turbo DNA-free kit	ThermoFisher Scientific (CH)	Cat# AM1907
SMARTer smRNA-Seq Kit for Illumina	Takara Bio Europe (FR)	Cat# 635029
QIAquick PCR Purification kit	Qiagen (DE)	Cat# 20104
NEBNext Ultra II DNA Library Prep Kit	New England BioLabs (USA)	Cat# E7645

**Deposited data**		

Sequencing raw data from the Funcional Genomic Center Zurich (FGCZ)	This paper; deposited to European Nucleotide Archive (ENA)	PRJEB76991
Tables S2 and S3 (https://data.mendeley.com/datasets/63rjyv54w4/2) with RNA sequencing and DRIP sequencing at CT6/18	This paper; deposited on Mendeley Data^42^	KRT6756a0b42be9a

**Experimental models: Cell lines**		

Nono gene trap Mouse Embrionic Fibroblasts (MEFs, C57Bl6 genotype)	University of California, Davis	Nono^gt^
PSPC1 gene trap Mouse Embrionic Fibroblasts (MEFs, C57Bl6 genotype)	University of California, Davis	PSPC1^gt^

**Oligonucleotides**		

See Table S1 for list of qPCR primers		

**Recombinant DNA**		

Lentiviral vector encoding the promoter region of mouse Bmal1 gene driving a luciferase reporter mBmal1-Luc	Gossan et al.^73^	Addgene, Cat# 182762
Lentiviral packaging plasmid psPAX2	Didier Trono Lab: Packaging and Envelope Plasmids (unpublished)	Addgene, Cat# 12260
Lentiviral envelope plasmid MD2.G	Didier Trono Lab: Packaging and Envelope Plasmids (unpublished)	Addgene, Cat# 12259
Lentiviral vector EGFP marker	Didier Trono Lab: Packaging and Envelope Plasmids (unpublished)	Addgene, Cat# 12254
pEGFP-N2-2XNLS-RNAse H1 delta 1-27 (WT)	Crossley ey al.^74^	Addgene, Cat# 196702

**Software and algorithms**		

LumiCycle Analysis software c2.31	Actimetrics	–
DiscoRhythm application v1.2.1	Carlucci et al.^75^	–
JunctionSeq v1.5.4	Hartley and Mullikin^39^	–
NRSA (Nascent RNA sequencing analysis) v2	Wang al.^76^	–
MACS2 (Model-based Analysis of ChIP-*seq)* v2.2.9.1	Zhang et al.^77^	–
ChIPSeeker v3.20	Yu et al.^78^	–
ImageJ software	Schindelin et al.^79^	_
Web application Statistic Kingdom 2017 for statistics	www.statskingdom.com	–
ShinyGO (Gene Ontology analysis) v0.77	Ge et al.^80^	–
Revigo (Gene Ontology) v1.8.1	Supek et al.^81^	–
Fisher Exact Test in R v4.0.5	R	–
STAR RNA-seq aligner	Dobin et al.^82^	–
RSEM for transcripts quantification from RNA-seq	Li and Dewey^83^	–
TMM to normalize expression from RNA-seq	Robinson and Oshlack^84^	–
Benjamini-Hochberg approach to FDR in multiple testing	Benjamini and Hochberg^85^	–
Burrows-Wheeler alignment for short sequenced reads	Li and Durbin^86^	–
BEDTools to compare genomic features	Quinlan and Hall^87^	–

### Experimental model and participant details

Animal procedures were carried out according to the veterinary law of the Canton of Zurich. The generation of the NONO^gt^ and PSPC1^gt^ mouse strains has been previously described.^1^ Chimeric mice were obtained from *Nono*^*gt*^ ES cells (C57Bl6 genotype) via standard blastocyst injection into SV129 mice by the University of California, Davis. Individual chimeric mice were back-crossed 4–10 generations against C57Bl6.

The strain was maintained on the C57BL/6J genetic background by backcrossing with wild-type mice from Janvier-Labs, France.

C57BL/6 female wild-type, NONO^gt^ or PSPC1^gt^ (12-13 days after mating), were anesthetized with isoflurane and killed by cervical dislocation. The positive Nono^gt^ mice were identified by ear genotyping.

Mouse embryonic fibroblasts (MEFs) were obtained as previously described by Qiu et al. and maintained in Dulbecco’s modified Eagle’s medium (DMEM) supplemented with 1% penicillin/streptomycin and 10% foetal bovine serum (FBS) supplemented with high glucose, L-glutamine, and phenol red.^88^ MEFs were cultured at 37°C in a humidified incubator with 5% CO_2_. The cells were passaged when they were approximately 80% confluent and used between the third and eighth passages.

Mycoplasma contamination was tested by a Mycoalert detection kit.

### Method details

#### Experiment 1: Cell synchronization

##### Experimental setup

MEFs were synchronized by adding a new culture medium supplemented with 100 nM of dexamethasone for 30 minutes, followed by extensive washing with PBS.

The effective synchronization by dexamethasone exposure was evaluated by the Real-Time Luminescence reporting of a circadian gene in the genetically modified MEFs. The cells were transfected with lentiviral particles, prepared by using the vector encoding the promoter region of mouse Bmal1 gene driving a luciferase reporter (Addgene, Plasmid #182762),^73^ the packaging plasmid psPAX2 (Addgene plasmid #12260 and the envelope plasmid MD2G (Addgene plasmid 12259) (pWPI for EGFP expression used as control, Addgene Plasmid #12254). For days later, after puromycin selection, the cells were exposed to dexamethasone 100 nM for 30 minutes and 100mM of Luciferin was added. The cell dishes were then put in a built-in-house apparatus (with constant darkness and controlled temperature) where photomultipliers detected the emitted luminescence for several days.

#### Experiment 2: Round-the-clock experiment

##### Experimental setup

For circadian transcriptome determination, the cells were collected every 4 hours starting 12 hours after the dexamethasone shock. All the samples were prepared in triplicate, and the extraction of RNA was accomplished by using the Total RNA Purification Micro Kit from Norgen Biotek, according to the protocol of the supplier.

#### Experiment 3: RNA seq at two time points

##### Experimental setup

MEFs were synchronized by adding a new culture medium supplemented with 100 nM dexamethasone for 30 minutes, followed by extensive washing with PBS. The cells were collected 18 hours (circadian time 18, CT18) and 30 hrs (circadian time 6, CT6) after dexamethasone treatment. All the samples were prepared in triplicate, and RNA was extracted using the Total RNA Purification Micro Kit from Norgen Biotek, Canada (cat. 35300), according to the protocol of the supplier.

#### Experiment 4: Cytoplasmic and nuclear RNA sequencing

##### Experimental setup

WT and NONO^gt^ MEFs were used 24 hours after dexamethasone shock (100 nM, 30 minutes). The experiment was carried out in triplicate.The cytoplasmic RNA was obtained as described by Suzuki et al.^89^ However, to better separate the nuclear RNA, we also used the sucrose cushion buffer method.

Total RNA preparation: after detaching the cells by Trypsin-EDTA and washing, the cell pellet was resuspended in 900 μl of cold PBS + 0.1% NP-40. Two hundred microliters of the suspension were immediately removed, and the total RNA was extracted by the Total RNA Purification Micro Kit from Norgen Biotek. For cytoplasmic RNA preparation, the remaining suspension was kept on ice for 5 minutes and then centrifuged for 30 seconds at 5000 g and 4°C. A total of 250 μl of the supernatant was taken, and the RNA was extracted. This fraction represents the cytoplasmic RNA. For nuclear RNA preparation, the remaining supernatant was discarded, and the pellet (nuclei) was resuspended in 500 μl of PBS + 0.1% NP-40. Afterward, the mixture was incubated for 10 minutes on ice and subsequently homogenized in a Dounce homogenizer. Three millilitres of 0.32 M sucrose buffer (0.32 M sucrose, 3 mM CaCl_2_, 2 mM Mg-acetate, 0.1 mM EDTA, 10 mM Tris-HCl (pH 8.0), 1 mM DTT, 0.5% NP-40) containing 1 μl/ml RNaseOUT and 3 ml of 2 M sucrose buffer were added. The suspension was stratified on 3 ml of sucrose buffer 2 M (+ RNAseOUT 1 μl/ml) and centrifuged at 13200 g for 45 minutes at 4°C. The nuclear RNA was extracted from the pellet by using the same kit.

#### Experiment 5: Global Run-On sequencing

##### Experimental setup

The experiment was performed as described by A. Gardini.^37^ Dishes of WT and NONO^gt^ MEFs were synchronized by dexamethasone treatment (1 μM for 20 minutes) and incubated for 18 hours (CT18) or 30 hours (CT6) after dexamethasone shock.

The collected cells passed through sequential steps of resuspensions in cold swelling buffer (10 mM Tris-HCl, pH 7.5; 2 mM MgCl_2_, 3 mM CaCl_2_); swelling buffer supplemented with 10% glycerol; and lysis buffer (swelling buffer supplemented with glycerol and 1% Igepal). The final nuclear pellet was resuspended in a freezing buffer at 1.5 × 10^7^ nuclei/100μl and kept at -80°C.

The nuclei Run-On staining was carried out by using 100 μl of frozen nuclei per sample, added to 100 μl of 2X Nuclear Run-On (NRO) buffer (10 mM Tris-HCl, pH 8; 5 mM MgCl_2_; 300 mM KCl; 1 mM DTT; 500 μM ATP; 500 μM GTP; 500 μM Br-UTP; 2 μM CTP; 200 U/ml Superase In; 1% N-Laurylsarcosine (sodium salt solution); and nuclease-free water). The samples were incubated at 30°C for 7 minutes, and total RNA was extracted using Trizol-LS reagent, for a final concentration of approximately 80 μg in 50 μl/sample in nuclease-free distilled water. The RNA was then treated with DNase (Turbo DNA-free Kit) for 30 minutes at 37°C, followed by fragmentation with fragmentation reagents (Invitrogen) at 70°C for 8 minutes. The samples were purified by using Micro Bio-Spin P-30 gel columns and treated twice with T4 polynucleotide kinase (PNK) for 60 minutes.

For immunoprecipitation, each RNA sample was brought to a 200 μl of binding buffer (0.25X SSPE, 0.05% Tween 20, 37.5 mM NaCl, 1 mM EDTA, SUPERase-In, nuclease free water) and added to anti-BrdU-conjugated agarose bead slurry (70 μl/sample), which was previously blocked and diluted in binding buffer. After 60 minutes of incubation at room temperature, the beads were sequentially washed at room temperature with binding buffer, low-salt buffer (0.2X SSPE, 0.05% Tween 20, 1 mM EDTA, and nuclease-free water), high-salt buffer (0.2X SSPE, 137.5 mM NaCl, 0.05% Tween 20, 1 mM EDTA, and nuclease-free buffer), TE buffer (0.01 M Tris, 0.001 M EDTA, pH 7.4) and Tween 20 (0.05%). The brominated RNA was eluted by incubation with 100 μl of elution buffer (50 mM Tris (pH 7.5), 150 mM NaCl, 0.1% SDS, 20 mM DTT, 1 mM EDTA, and nuclease-free water) for 10 minutes at 37°C, after which the elution was repeated 3 additional times for a total volume of 400 μl. The RNA was purified by ethanol precipitation, washed and resuspended in 7 μl of nuclease-free water with 0.05% Tween 20 and 1 U/μl of SUPERase-In. The final recovery was 2.5-3 ng/μl.

#### Experiment 6: DRIP-sequencing (DNA-RNA immunoprecipitation followed by high-throughput DNA sequencing)

##### Experimental setup

The experiment was carried out according to the protocol described by Sanz L and Chédin F.^41^ Briefly, the steps were as follows.

For cell lysis, WT and NONO^gt^ MEFs (10^7^ cells) at 80% confluency were resuspended in TE buffer (10 mM Tris, pH 8.0; 1 mM EDTA) supplemented with SDS (final concentration of 0.6%) and 5 μl of 20 mg/mL proteinase K. The tube was inverted several times until viscosity appeared, after which the mixture was incubated overnight at 37°C. The DNA lysate was cleaned by phenol/chloroform/isoamyl alcohol extraction in a high-density Maxtract phase-lock gel tube, followed by precipitation with 2.5 volumes of 100% ethanol and 0.1 volume of 3 M NaOAc, pH 5.2. The white precipitated DNA was removed, washed twice with 80% ethanol, and resuspended in TE buffer.

The DNA was then fragmented by incubating overnight at 37°C with a cocktail of restriction enzymes, BsrGI, EcoRI, HindIII, SspI, and XbaI (50 U each), in combination with NEB CUT SMART Buffer 1X, 0.1 mg/ml BSA and 1 mM spermidine. The digested DNA was cleaned with phenol/chloroform/isoamyl alcohol (25:24:1) in a 2-mL phase-lock gel light tube and precipitated by centrifuging with ethanol, NaOA and glycogen. The washed DNA was resuspended in 100 μl of TE buffer, and the concentration was measured via a NanoDrop spectrophotometer. As a control for specificity, an aliquot of DNA (10 μg) was treated with 5 μl of RNase H for 6 hours at 37°C to remove the DNA‒RNA hybrids.

For each sample, 8 μg of digested DNA (treated or not with RNase H) was immunoprecipitated with 10 μl of the S9.6 antibody in DRIP binding buffer (sodium phosphate 100 mM pH 7.0, NaCl 1.4 M, Triton X-100 0.5%) by incubating overnight at 4°C with slow rotation. A protein A-agarose bead slurry (70 μl/sample, previously washed) was added to the DNA, which was subsequently incubated for 2 hours at 4°C with slow rotation. The beads were then washed three times with DRIP binding buffer for 15 minutes on a rotator at room temperature, and the nucleic acids were eluted with 100 μl of DRIP elution buffer (Tris 50 mM pH 8.0, EDTA 10 mM pH 8.0, SDS 0.5%) and 5 μl of proteinase K (20 mg/mL) at 55°C for 45 minutes. The beads were then spun down, and the DNA in the supernatant was purified with a Qiagen PCR Purification Kit, and eluted in a final elution volume of 30 μl.

#### Experiment 7: DNA damage evaluation by immunofluorescence

##### Experimental setup

MEFs were grown on glass bottom Ibidi 12-well chambers, coated with fibronectin.

The cells were treated with ARV-825 30 nM or DMSO vehicle for six hours.

In a side experiment, the cells were transiently transfected with the pEGFP-N2-2XNLS-RNAse H1 delta plasmid (0.15 μg/well) and 48 hours later treated with ARV-825. pEGFP-N2-2XNLS-RNaseH1 delta 1-27 (WT) was a gift from Karlene Cimprich’s lab (Addgene plasmid # 196702; RRID:Addgene_196702).^74^

After washing, the cells were fixed in paraformaldehyde for 20 minutes and permeabilized with PBS/0.1 % Triton-X100 for 15 minutes. The cells were then blocked with Normal Goat Serum in PBS/0.1% Tween-20 for 1 hour. The immunofluorescence was performed by anti-RNA/DNA hybrids S9.6 from mouse (1:200) and pAB to γ-H2AX (phospho S139) from rabbit (1:1000) overnight at 4°C. After washing with PBS/0.1% tween-20, the cells were stained with secondary antibodies Alexa Fluor 488 anti-mouse (green) and Alexa Fluor 594 anti-rabbit (red) (both 1:1000), for one hour at room temperature. The cells were counterstained with DAPI (1μg/ml) for 7 minutes, washed, and mounted by Dako mounting medium.

### Quantification and statistical analysis

The quantification, the statistical analyses, and the software programs are described in detail in this section as well as summarized in the figure legends.

#### Experiment 1: Cell synchronization

The cell autonomous clock period was obtained by using the LumiCycle software (from Actimetrics), with an automatic subtraction of the baseline. The period values are reported in the related figure legend.

#### Experiment 2: Round-the-clock experiment

The samples were deep sequenced at the Functional Genomic Centre Zürich (FGCZ). The library was prepared by Illumina TruSeq mRNA Library Prep with polyA selection.

The samples were sequenced on Illumina HiSeq4000. The single-end reads of length 125 were aligned by STAR to the mouse genome GRCm38.p5, and the genes were quantified by the RSEM tool using the same reference as for STAR.^82^^,^^83^ The normalized counts were generated via the TMM method implemented in EdgeR v3.32.1.^84^

For the analysis of gene rhythmicity (n=3 biological sample for each time point), the phase, amplitude, and statistical significance of circadian gene expression were determined by the DiscoRhythm application v1.2.1,^75^ which sequentially performed outlier detection, identified the dominant periodicities in the data, and, finally, detected the rhythmicity. The sample similarity was assessed by the Pearson method (threshold ∼3 standard deviations). This analysis indicated one replicate at CT24 as an outlier and it was removed from downstream analysis. The periodogram showed 24 hours as the dominant periodicity, and rhythmicity was detected by using Cosinor and JTK analyses (P<0.05).^29^^,^^30^

The analysis of rhythmicity is also in the figure legends.

#### Experiment 3: RNA seq at two time points

The samples were deep sequenced at the Functional Genomic Centre Zürich (FGCZ). The library was prepared by Illumina TruSeq mRNA Library Prep, with polyA selection.

The samples were sequenced on Illumina NovaSeq 6000 platform. The single-end reads of length 100 were aligned by STAR to the mouse genome GRCm39, and the genes were quantified by Kallisto using the same reference as for STAR.^82^ The normalized counts were generated via the TMM method implemented in EdgeR v3.32.1.^84^ The FDR for differential expression analysis was calculated with the Benjamini–Hochberg method.^85^ Upregulated and downregulated genes were called at an absolute log2 fold change larger than one and with FDR cut-off at 0.01.

The significance of the overlap of differentially expressed genes in the different conditions and time points are analysed by the Fisher’s exact test, performed using R v4.0.5, with the R Stats Package. The null hypothesis (independent two groups) is that the odds ratio is no larger than 1. The alternative is that the odds ratio is larger than 1.0. The odds ratio and p-values are in the figure legends.

The alternative exon and splice junction usage was analysed by the R package JunctionSeq v1.5.4.^39^ From the data generated in the high-throughput RNA-seq at the two time points, the software detects the differential usage of exons (or exon regions) and splice junctions, relative to the whole expression of a gene. For each exon, it calculates the ratio between the number of reads mapping to this exon and the reads mapping to all the exons of the same gene. If the ratio changes across conditions, a differential usage of the exon is deduced (threshold of FDR<0.01). A further analysis is also carried out at the gene level, testing the hypothesis that one or more exons or junctions of the gene are differentially expressed. The differential exon usage can be considered a proxy for the evaluation of alternative isoforms (FDR<0.05).

The differential exon usage was validated by qPCR: A total of 1 μg of cDNA was analysed via quantitative PCR with HOT FIREPol EvaGreen qPCR Mix Plus (ROX) using the primers at a concentration of 1 μM.

Primers sequences are reported in Table S1.

The relative amounts of input recovery were calculated by the formula 100∗2ˆ((CT input-log2 dilution factor)-CT sample). The unpaired t-test was performed by using Statistics Kingdom 2017 (web application), available at http://www.statskingdom.com. Significant p-value < 0.05, also shown in the figure legend.

#### Experiments 2, 3, and 5: Gene ontology analyses

Overrepresented G.O. Biological processes were analysed and visualized by ShinyGO v0.77, using the expressed genes as background.^80^ Enrichment analysis was performed based on the hypergeometric distribution followed by false discovery rate (FDR) correction. Overrepresented GO terms were selected by FDR (cutoff at 0.05) and ranked by fold enrichment. When the GO terms are summarized, we used Revigo v1.8.1, where the GO terms are represented by bubbles. The colour intensity of the bubbles corresponds to the adjusted p-value (dark colour for lower p-adjusted value). The size of the bubble corresponds to the LogSize value for the GO term.^81^

The levels of significance of GO terms from Shiny are also shown in the figure legends.

#### Experiment 4: Cytoplasmic and nuclear RNA sequencing

The cytoplasmic and nuclear RNA samples (n=3 biological replicates) were pooled before sequencing at the Functional Genomic Centre Zürich (FGCZ). The library preparation was performed using an Illumina TruSeq total RNA Library Prep kit with ribosomal depletion. Sequencing was performed on an Illumina HiSeq2500 platform. After trimming, the paired-end reads of length 125 were aligned by Star to the mouse genome GRCm38.p5, and the genes were quantified by the function countOverlaps in the R package Genomic Features. The normalized counts were generated via the TMM method implemented in EdgeR v3.32.1.^84^ The FDR for differential expression analysis was calculated with the Benjamini–Hochberg method.^85^ Upregulated and downregulated genes were defined based on an absolute log_2_ fold change greater than one and with FDR cutoff at 0.01.

The degree of DEGs in the nucleus and cytoplasm of Nono^gt^ compared to WT MEFs was analysed by the Fisher’s exact test, performed using R v4.0.5, with the R Stats Package. The null hypothesis (independent two groups) is that the odds ratio is no larger than 1. The alternative is that the odds ratio is larger than 1.0. The odds ratio and p-values are in the figure legends.

Validation of the cytoplasm and nuclei fractionation by qPCR: a total of 1 μg of cDNA was analysed via quantitative PCR with HOT FIREPol EvaGreen qPCR Mix Plus (ROX) using the primers at a concentration of 0.3 μM. The primers are reported in Table S1 (MEN epsilon/beta primers are designed according to Sunwoo et al.^90^

The fold gene expression was calculated by the formula 2ˆ-(ΔΔCt).

The unpaired t-test was performed by using Statistics Kingdom 2017 (web application), available at http://www.statskingdom.com. Significant p-value < 0.05, also shown in the figure legend.

#### Experiment 5: Global Run-On sequencing

PNK treatment of the 3′ end of the OH group made the RNA fragments suitable for artificial polyadenylation before adapter binding, as described in the protocol of the SMARTer smRNA-Seq Kit for Illumina (Takarabio), which we used for the preparation of the stranded library. PCR amplification was performed for 17 cycles. The control of the dimensions of the library fragments was performed by using Tape Station at the genomic center; the peaks obtained were approximately 240 bp, within the optimal size range indicated by Gardini paper (200-250 bp).^37^

Sequencing was performed on an Illumina HiSeq2500 platform. One of the WT 18 weeks old mice (sample CT18WT-C_pool1) had to be discarded from the analysis because of the low number of reads obtained from the sequencing.

After the adapter trimming and low-quality sequence removal, only reads which are > 32 bp in length were retained and aligned by Burrows–Wheeler Aligner (BWA) with the reference genome mm10.^86^ Aligned files in bam format were used as input into the software NRSA v2 (nascent *RNA* sequencing analysis) with default parameters.^76^

For known genes, NRSA performs the quantification of RNA polymerase abundance on promoter-proximal (pp) and gene-body (gb) regions, and calculation of pausing index. A gene is determined transcriptionally active if its promoter-proximal density is greater than zero and the gene-body density is greater than 4 reads/kb after total read count normalization. Transcriptional changes were calculated for all active genes at multiple stages, including initiation, elongation, and pausing. The condition-dependent transcriptional changes and pausing index alterations were called at an absolute log2 fold change larger than one and with p-adj for multiple comparisons cut-off at 0.05.

The correlations between the gene body transcription and proximal transcription or pausing index were done by using Statistics Kingdom 2017 (web application), available from http://www.statskingdom.com. The r and p-values are shown in the figure legend.

The overlap at CT6 and CT18 of the genes with differences in gene body (elongation) or pausing near the promoter was analysed by the Fisher’s exact test, performed using R v4.0.5, with the R Stats Package. The null hypothesis (independent two groups) is that the odds ratio is no larger than 1. The alternative is that the odds ratio is larger than 1.0. The odds ratio and p-values are in the figure legends.

#### Experiment 6: DRIP-sequencing (DNA-RNA immunoprecipitation followed by high-throughput DNA sequencing)

The samples were delivered to the Functional Genomic Centre Zürich (FGCZ) for further fragmentation, Illumina library preparation, and sequencing. The NEBNext Ultra II DNA Library Prep Kit was used for library preparation. Sequencing was performed on an Illumina NovaSeq 6000 platform. After trimming the adapter and low-quality bases, paired-end reads 100 in length were aligned to the mouse genome GRCm39 with Bowtie. Peaks were called for each replicate relative to the input through MACS2 with the following parameters: no-model, broad.^77^ The peaks of all the samples were combined as bed files. This file was sorted by the location in the genome (bedtools sort), and the overlapping peaks across different samples were combined by bedtools merge, keeping the information from which the peak was derived.^87^ A list of consensus peaks was obtained by keeping all the peaks detected in at least two samples. The peaks were annotated by ChIPSeeker^78^ with a GTF file obtained from the GENCODE release M42. The differentially expressed peaks (gains and losses) were called by EdgeR v3.32.1 according to an absolute log_2_ fold change greater than one and an FDR cutoff at 0.01.

To analyse the variation of gene expression as a function of R-loops gain or loss, at the CT6 and CT18, the Pearson Correlation Coefficient was calculated by using Statistics Kingdom 2017 (web application), available at http://www.statskingdom.com.

The significance of the coefficient was evaluated according to Cohen’s guidelines (Cohen J., 1988, Statistical power analysis for the behavioral sciences, 2nd ed., p.413)^42^: |r| < 0.1 very small correlation effect size; 0.1 ≤ |r| < 0.3 small correlation effect size. The analysis is summarized in the figure legend.

DRIP was validated by means of qPCR: a total of 1 μg of cDNA was analysed via quantitative PCR with HOT FIREPol EvaGreen qPCR Mix Plus (ROX) using the primers at a concentration of 1 μM.

Primers sequences are reported in Sanz L and Chédin F^41^ and in Table S1.

The relative amounts of input recovery were calculated by the formula 100∗2ˆ((CT input-log2 dilution factor)-CT sample). The unpaired t-test was performed by using Statistics Kingdom 2017 (web application), available at http://www.statskingdom.com. Significant p-value < 0.001, also shown in the figure legend.

#### Experiment 7: DNA damage evaluation by immunofluorescence

The images were taken by Zeiss AiryScan 800 and ImageJ software was used for quantitative analysis.^79^ The nuclei were selected as regions of interest (ROI) using the DAPI channel and only the co-localizing regions in the other two channels were used to integrate the nuclear fluorescence intensity.

The data were log transformed to get normal distribution (evaluated by Shapiro-Wilk test, significance level p > 0.05) and equal variances (two-tailed F test for two groups or Levine test for more groups, significance level p > 0.05) The two-way ANOVA test checked the existence of differences among the means of different groups (significance level p < 0.05), and whether interaction between genotype and treatment exists (significance level p < 0.05). The Tukey HSD post-hoc test for multiple comparison was used for testing of main effects (significance level p < 0.05).

For the side experiment with RNaseH1 transfection, the nuclear integrated signals from cells with and without EGFP expression in the same images were compared by two-tailed unpaired t-test. with significance level p < 0.05.

All the statistical test for this experiment were performed by using Statistics Kingdom 2017 (web application), available from http://www.statskingdom.com.

The analyses are also summarized in the figure legends, with the obtained results.

## References

[1] Kowalska E., Ripperger J.A., Hoegger D.C., Bruegger P., Buch T., Birchler T., Mueller A., Albrecht U., Contaldo C., Brown S.A. (2013). NONO couples the circadian clock to the cell cycle. Proc. Natl. Acad. Sci. USA.

[2] Benegiamo G., Mure L.S., Erikson G., Le H.D., Moriggi E., Brown S.A., Panda S. (2018). The RNA-Binding Protein NONO Coordinates Hepatic Adaptation to Feeding. Cell Metab..

[3] Mircsof D., Langouët M., Rio M., Moutton S., Siquier-Pernet K., Bole-Feysot C., Cagnard N., Nitschke P., Gaspar L., Žnidarič M. (2015). Mutations in NONO lead to syndromic intellectual disability and inhibitory synaptic defects. Nat. Neurosci..

[4] Feng P., Li L., Deng T., Liu Y., Ling N., Qiu S., Zhang L., Peng B., Xiong W., Cao L. (2020). NONO and tumorigenesis: More than splicing. J. Cell Mol. Med..

[5] Knott G.J., Bond C.S., Fox A.H. (2016). The DBHS proteins SFPQ, NONO and PSPC1: a multipurpose molecular scaffold. Nucleic Acids Res..

[6] Ishitani K., Yoshida T., Kitagawa H., Ohta H., Nozawa S., Kato S. (2003). p54nrb acts as a transcriptional coactivator for activation function 1 of the human androgen receptor. Biochem. Biophys. Res. Commun..

[7] Amelio A.L., Miraglia L.J., Conkright J.J., Mercer B.A., Batalov S., Cavett V., Orth A.P., Busby J., Hogenesch J.B., Conkright M.D. (2007). A coactivator trap identifies NONO (p54 nrb) as a component of the cAMP-signaling pathway. Proc. Natl. Acad. Sci. USA.

[8] Dong X., Yu C., Shynlova O., Challis J.R.G., Rennie P.S., Lye S.J. (2009). p54nrb Is a Transcriptional Corepressor of the Progesterone Receptor that Modulates Transcription of the Labor-Associated Gene, Connexin 43 (Gja1). Mol. Endocrinol..

[9] Kowalska E., Ripperger J.A., Muheim C., Maier B., Kurihara Y., Fox A.H., Kramer A., Brown S.A. (2012). Distinct Roles of DBHS Family Members in the Circadian Transcriptional Feedback Loop. Mol. Cell Biol..

[10] Cristini A., Groh M., Kristiansen M.S., Gromak N. (2018). RNA/DNA Hybrid Interactome Identifies DXH9 as a Molecular Player in Transcriptional Termination and R-Loop-Associated DNA Damage. Cell Rep..

[11] Mosler T., Conte F., Longo G.M.C., Mikicic I., Kreim N., Möckel M.M., Petrosino G., Flach J., Barau J., Luke B. (2021). R-loop proximity proteomics identifies a role of DDX41 in transcription-associated genomic instability. Nat. Commun..

[12] Wang I.X., Grunseich C., Fox J., Burdick J., Zhu Z., Ravazian N., Hafner M., Cheung V.G. (2018). Human proteins that interact with RNA/DNA hybrids. Genome Res..

[13] Boleslavska B., Oravetzova A., Shukla K., Nascakova Z., Ibini O.N., Hasanova Z., Andrs M., Kanagaraj R., Dobrovolna J., Janscak P. (2022). DDX17 helicase promotes resolution of R-loop-mediated transcription–replication conflicts in human cells. Nucleic Acids Res..

[14] Santos-Pereira J.M., Aguilera A. (2015). R loops: new modulators of genome dynamics and function. Nat. Rev. Genet..

[15] Niehrs C., Luke B. (2020). Regulatory R-loops as facilitators of gene expression and genome stability. Nat. Rev. Mol. Cell Biol..

[16] Costantino L., Koshland D. (2015). The Yin and Yang of R-loop biology. Curr. Opin. Cell Biol..

[17] García-Muse T., Aguilera A. (2019). R Loops: From Physiological to Pathological Roles. Cell.

[18] Chen Y.-Z., Bennett C.L., Huynh H.M., Blair I.P., Puls I., Irobi J., Dierick I., Abel A., Kennerson M.L., Rabin B.A. (2004). DNA/RNA Helicase Gene Mutations in a Form of Juvenile Amyotrophic Lateral Sclerosis (ALS4). Am. J. Hum. Genet..

[19] Elsakrmy N., Cui H. (2023). R-Loops and R-Loop-Binding Proteins in Cancer Progression and Drug Resistance. Int. J. Mol. Sci..

[20] Bhatia V., Herrera-Moyano E., Aguilera A., Gómez-González B. (2017). The Role of Replication-Associated Repair Factors on R-Loops. Genes.

[21] Petti E., Buemi V., Zappone A., Schillaci O., Broccia P.V., Dinami R., Matteoni S., Benetti R., Schoeftner S. (2019). SFPQ and NONO suppress RNA:DNA-hybrid-related telomere instability. Nat. Commun..

[22] Klaric J.A., Wüst S., Panier S. (2021). New Faces of old Friends: Emerging new Roles of RNA-Binding Proteins in the DNA Double-Strand Break Response. Front. Mol. Biosci..

[23] Wang Y.-L., Zhao W.-W., Bai S.-M., Ma Y., Yin X.-K., Feng L.-L., Zeng G.-D., Wang F., Feng W.-X., Zheng J. (2022). DNA damage-induced paraspeckle formation enhances DNA repair and tumor radioresistance by recruiting ribosomal protein P0. Cell Death Dis..

[24] Deshar R., Yoo W., Cho E.-B., Kim S., Yoon J.-B. (2019). RNF8 mediates NONO degradation following UV-induced DNA damage to properly terminate ATR-CHK1 checkpoint signaling. Nucleic Acids Res..

[25] Montecucco A., Biamonti G. (2013). Pre-mRNA processing factors meet the DNA damage response. Front. Genet..

[26] Zhang S., Cooper J.A., Chong Y.S., Naveed A., Mayoh C., Jayatilleke N., Liu T., Amos S., Kobelke S., Marshall A.C. (2023). NONO enhances mRNA processing of super-enhancer-associated GATA2 and HAND2 genes in neuroblastoma. EMBO Rep..

[27] Iino K., Mitobe Y., Ikeda K., Takayama K.I., Suzuki T., Kawabata H., Suzuki Y., Horie-Inoue K., Inoue S. (2020). RNA-binding protein NONO promotes breast cancer proliferation by post-transcriptional regulation of SKP2 and E2F8. Cancer Sci..

[28] Lee K., Jang S.H., Tian H., Kim S.J. (2020). NonO Is a Novel Co-factor of PRDM1 and Regulates Inflammatory Response in Monocyte Derived-Dendritic Cells. Front. Immunol..

[29] Cornelissen G. (2014). Cosinor-based rhythmometry. Theor. Biol. Med. Model..

[30] Hughes M.E., Hogenesch J.B., Kornacker K. (2010). JTK_CYCLE: An Efficient Nonparametric Algorithm for Detecting Rhythmic Components in Genome-Scale Data Sets. J. Biol. Rhythms.

[31] Takeuchi A., Iida K., Tsubota T., Hosokawa M., Denawa M., Brown J.B., Ninomiya K., Ito M., Kimura H., Abe T. (2018). Loss of Sfpq Causes Long-Gene Transcriptopathy in the Brain. Cell Rep..

[32] Bond C.S., Fox A.H. (2009). Paraspeckles: nuclear bodies built on long noncoding RNA. J. Cell Biol..

[33] Ingram H.B., Fox A.H. (2024). Unveiling the intricacies of paraspeckle formation and function. Curr. Opin. Cell Biol..

[34] Kaneko S., Rozenblatt-Rosen O., Meyerson M., Manley J.L. (2007). The multifunctional protein p54nrb/PSF recruits the exonuclease XRN2 to facilitate pre-mRNA 3′ processing and transcription termination. Genes Dev..

[35] Lewis B.A., Das S.K., Jha R.K., Levens D. (2023). Self-assembly of promoter DNA and RNA Pol II machinery into transcriptionally active biomolecular condensates. Sci. Adv..

[36] Core L.J., Waterfall J.J., Lis J.T. (2008). Nascent RNA Sequencing Reveals Widespread Pausing and Divergent Initiation at Human Promoters. Science (1979).

[37] Gardini A. (2017). Global Run-On Sequencing (GRO-Seq). Methods Mol. Biol..

[38] Kameoka S., Duque P., Konarska M.M. (2004). p54nrb associates with the 5′ splice site within large transcription/splicing complexes. EMBO J..

[39] Hartley S.W., Mullikin J.C. (2016). Detection and visualization of differential splicing in RNA-Seq data with JunctionSeq. Nucleic Acids Res..

[40] Cong S., Di X., Li R., Cao Y., Jin X., Tian C., Zhao M., Wang K. (2022). RBM10 regulates alternative splicing of lncRNA Neat1 to inhibit the invasion and metastasis of NSCLC. Cancer Cell Int..

[41] Sanz L.A., Chédin F. (2019). High-resolution, strand-specific R-loop mapping via S9.6-based DNA–RNA immunoprecipitation and high-throughput sequencing. Nat. Protoc..

[42] Moriggi E., Pisteljic M., Rosi-Andersen A., Opitz L., Azzi A., Brown S.A. (2025). Data for: The NONO protein regulates nonclassical DNA structure: effects on circadian genes and DNA damage. Mendeley Data.

[43] Cohen J. (2013).

[44] Castillo-Guzman D., Chédin F. (2021). Defining R-loop classes and their contributions to genome instability. DNA Repair.

[45] Grunseich C., Wang I.X., Watts J.A., Burdick J.T., Guber R.D., Zhu Z., Bruzel A., Lanman T., Chen K., Schindler A.B. (2018). Senataxin Mutation Reveals How R-Loops Promote Transcription by Blocking DNA Methylation at Gene Promoters. Mol. Cell.

[46] Skourti-Stathaki K., Torlai Triglia E., Warburton M., Voigt P., Bird A., Pombo A. (2019). R-Loops Enhance Polycomb Repression at a Subset of Developmental Regulator Genes. Mol. Cell.

[47] Lam F.C., Kong Y.W., Huang Q., Vu Han T.-L., Maffa A.D., Kasper E.M., Yaffe M.B. (2020). BRD4 prevents the accumulation of R-loops and protects against transcription–replication collision events and DNA damage. Nat. Commun..

[48] EMILI A., SHALES M., McCRACKEN S., XIE W., TUCKER P.W., KOBAYASHI R., BLENCOWE B.J., INGLES C.J. (2002). Splicing and transcription-associated proteins PSF and p54nrb/NonO bind to the RNA polymerase II CTD. RNA.

[49] Jonkers I., Kwak H., Lis J.T. (2014). Genome-wide dynamics of Pol II elongation and its interplay with promoter proximal pausing, chromatin, and exons. Elife.

[50] Hodroj D., Recolin B., Serhal K., Martinez S., Tsanov N., Abou Merhi R., Maiorano D. (2017). An ATR-dependent function for the Ddx19 RNA helicase in nuclear R-loop metabolism. EMBO J..

[51] Chakraborty P., Huang J.T.J., Hiom K. (2018). DHX9 helicase promotes R-loop formation in cells with impaired RNA splicing. Nat. Commun..

[52] Gatti V., De Domenico S., Melino G., Peschiaroli A. (2023). Senataxin and R-loops homeostasis: multifaced implications in carcinogenesis. Cell Death Discov..

[53] Hatchi E., Skourti-Stathaki K., Ventz S., Pinello L., Yen A., Kamieniarz-Gdula K., Dimitrov S., Pathania S., McKinney K.M., Eaton M.L. (2015). BRCA1 Recruitment to Transcriptional Pause Sites Is Required for R-Loop-Driven DNA Damage Repair. Mol. Cell.

[54] de Amorim J.L., Leung S.W., Haji-Seyed-Javadi R., Hou Y., Yu D.S., Ghalei H., Khoshnevis S., Yao B., Corbett A.H. (2024). The putative RNA helicase DDX1 associates with the nuclear RNA exosome and modulates RNA/DNA hybrids (R-loops). J. Biol. Chem..

[55] Villarreal O.D., Mersaoui S.Y., Yu Z., Masson J.-Y., Richard S. (2020). Genome-wide R-loop analysis defines unique roles for DDX5, XRN2, and PRMT5 in DNA/RNA hybrid resolution. Life Sci. Alliance.

[56] Lim Y.W., Sanz L.A., Xu X., Hartono S.R., Chédin F. (2015). Genome-wide DNA hypomethylation and RNA:DNA hybrid accumulation in Aicardi–Goutières syndrome. Elife.

[57] Al-Hadid Q., Yang Y. (2016). R-loop: an emerging regulator of chromatin dynamics. Acta Biochim. Biophys. Sin..

[58] Yuan Y., Chen Q., Brovkina M., Clowney E.J., Yadlapalli S. (2024). Clock-dependent chromatin accessibility rhythms regulate circadian transcription. PLoS Genet..

[59] Kuhnert A., Schmidt U., Monajembashi S., Franke C., Schlott B., Grosse F., Greulich K.O., Saluz H.P., Hänel F. (2012). Proteomic identification of PSF and p54(nrb) as topBP1-interacting proteins. J. Cell. Biochem..

[60] Alfano L., Costa C., Caporaso A., Altieri A., Indovina P., Macaluso M., Giordano A., Pentimalli F. (2016). NONO regulates the intra-S-phase checkpoint in response to UV radiation. Oncogene.

[61] Udayakumar D., Dynan W.S. (2015). Characterization of DNA binding and pairing activities associated with the native SFPQ·NONO DNA repair protein complex. Biochem. Biophys. Res. Commun..

[62] Jaafar L., Li Z., Li S., Dynan W.S. (2017). SFPQ·NONO and XLF function separately and together to promote DNA double-strand break repair via canonical nonhomologous end joining. Nucleic Acids Res..

[63] Mamontova V., Trifault B., Burger K. (2024). Nono induces Gadd45b to mediate DNA repair. Life Sci. Alliance.

[64] Trifault B., Mamontova V., Cossa G., Ganskih S., Wei Y., Hofstetter J., Bhandare P., Baluapuri A., Nieto B., Solvie D. (2024). Nucleolar detention of NONO shields DNA double-strand breaks from aberrant transcripts. Nucleic Acids Res..

[65] Laspata N., Kaur P., Mersaoui S.Y., Muoio D., Liu Z.S., Bannister M.H., Nguyen H.D., Curry C., Pascal J.M., Poirier G.G. (2023). PARP1 associates with R-loops to promote their resolution and genome stability. Nucleic Acids Res..

[66] Krietsch J., Caron M.-C., Gagné J.-P., Ethier C., Vignard J., Vincent M., Rouleau M., Hendzel M.J., Poirier G.G., Masson J.-Y. (2012). PARP activation regulates the RNA-binding protein NONO in the DNA damage response to DNA double-strand breaks. Nucleic Acids Res..

[67] Li S., Li Z., Shu F.-J., Xiong H., Phillips A.C., Dynan W.S. (2014). Double-strand break repair deficiency in NONO knockout murine embryonic fibroblasts and compensation by spontaneous upregulation of the PSPC1 paralog. Nucleic Acids Res..

[68] Kolinjivadi A.M., Chong S.T., Ngeow J. (2021). Molecular connections between circadian rhythm and genome maintenance pathways. Endocr. Relat. Cancer.

[69] Kang T.-H., Leem S.-H. (2014). Modulation of ATR-mediated DNA damage checkpoint response by cryptochrome 1. Nucleic Acids Res..

[70] Gil Rodríguez, S. (2019). Functional Characterization of the Connection between the Circadian Clock and the DNA Damage and Repair Response in Arabidopsis thaliana.

[71] Dettori L.G., Torrejon D., Chakraborty A., Dutta A., Mohamed M., Papp C., Kuznetsov V.A., Sung P., Feng W., Bah A. (2021). A Tale of Loops and Tails: The Role of Intrinsically Disordered Protein Regions in R-Loop Recognition and Phase Separation. Front. Mol. Biosci..

[72] Fan X.-J., Wang Y.-L., Zhao W.-W., Bai S.-M., Ma Y., Yin X.-K., Feng L.-L., Feng W.-X., Wang Y.-N., Liu Q. (2021). NONO phase separation enhances DNA damage repair by accelerating nuclear EGFR-induced DNA-PK activation. Am. J. Cancer Res..

[73] Gossan N., Zeef L., Hensman J., Hughes A., Bateman J.F., Rowley L., Little C.B., Piggins H.D., Rattray M., Boot-Handford R.P., Meng Q.J. (2013). The Circadian Clock in Murine Chondrocytes Regulates Genes Controlling Key Aspects of Cartilage Homeostasis. Arthritis Rheum..

[74] Crossley M.P., Song C., Bocek M.J., Choi J.-H., Kousouros J.N., Sathirachinda A., Lin C., Brickner J.R., Bai G., Lans H. (2023). R-loop-derived cytoplasmic RNA–DNA hybrids activate an immune response. Nature.

[75] Carlucci M., Kriščiūnas A., Li H., Gibas P., Koncevičius K., Petronis A., Oh G. (2020). DiscoRhythm: an easy-to-use web application and R package for discovering rhythmicity. Bioinformatics.

[76] Wang J., Zhao Y., Zhou X., Hiebert S.W., Liu Q., Shyr Y. (2018). Nascent RNA sequencing analysis provides insights into enhancer-mediated gene regulation. BMC Genom..

[77] Zhang Y., Liu T., Meyer C.A., Eeckhoute J., Johnson D.S., Bernstein B.E., Nusbaum C., Myers R.M., Brown M., Li W., Liu X.S. (2008). Model-based Analysis of ChIP-Seq (MACS). Genome Biol..

[78] Yu G., Wang L.-G., He Q.-Y. (2015). ChIPseeker: an R/Bioconductor package for ChIP peak annotation, comparison and visualization. Bioinformatics.

[79] Schindelin J., Arganda-Carreras I., Frise E., Kaynig V., Longair M., Pietzsch T., Preibisch S., Rueden C., Saalfeld S., Schmid B. (2012). Fiji: an open-source platform for biological-image analysis. Nat. Methods.

[80] Ge S.X., Jung D., Yao R. (2020). ShinyGO: a graphical gene-set enrichment tool for animals and plants. Bioinformatics.

[81] Supek F., Bošnjak M., Škunca N., Šmuc T. (2011). REVIGO Summarizes and Visualizes Long Lists of Gene Ontology Terms. PLoS One.

[82] Dobin A., Davis C.A., Schlesinger F., Drenkow J., Zaleski C., Jha S., Batut P., Chaisson M., Gingeras T.R. (2013). STAR: ultrafast universal RNA-seq aligner. Bioinformatics.

[83] Li B., Dewey C.N. (2011). RSEM: accurate transcript quantification from RNA-Seq data with or without a reference genome. BMC Bioinf..

[84] Robinson M.D., Oshlack A. (2010). A scaling normalization method for differential expression analysis of RNA-seq data. Genome Biol..

[85] Benjamini Y., Hochberg Y. (1995). Controlling the False Discovery Rate: A Practical and Powerful Approach to Multiple Testing. J R Stat Soc Series B Stat Methodol.

[86] Li H., Durbin R. (2009). Fast and accurate short read alignment with Burrows–Wheeler transform. Bioinformatics.

[87] Quinlan A.R., Hall I.M. (2010). BEDTools: a flexible suite of utilities for comparing genomic features. Bioinformatics.

[88] Qiu L.Q., Lai W.S., Stumpo D.J., Blackshear P.J. (2016). Mouse Embryonic Fibroblast Cell Culture and Stimulation. Bio. Protoc..

[89] Suzuki K., Bose P., Leong-Quong R.Y., Fujita D.J., Riabowol K. (2010). REAP: A two minute cell fractionation method. BMC Res. Notes.

[90] Sunwoo H., Dinger M.E., Wilusz J.E., Amaral P.P., Mattick J.S., Spector D.L. (2009). MEN ε/β nuclear-retained non-coding RNAs are up-regulated upon muscle differentiation and are essential components of paraspeckles. Genome Res..

