# Supplementary material for: The NONO protein regulates nonclassical DNA structure: Effects on circadian genes and DNA damage
Source: iScience. 2025 Apr 11;28(5):112408. doi: 10.1016/j.isci.2025.112408 (PMC12063141; doi:10.1016/j.isci.2025.112408)
Supplement: Document S1. Figures S1–S12 and Table S1 [file mmc1.pdf]

iScience, Volume *28*

## Supplemental information

# The NONO protein regulates nonclassical DNA structure: Effects on circadian genes and DNA damage

**Ermanno Moriggi, Melissa Pisteljic, Alex Rosi-Andersen, Lennart Opitz, Abdelhalim Azzi, and Steven A. Brown**

**Supplemental Information (Document S1)**

**SUPPLEMENTAL FIGURES 1-12 WITH FIGURE TITLES AND FIGURE LEGENDS**

**SUPPLEMENTAL TABLE 1**

# Figure S1

Synchronized WT

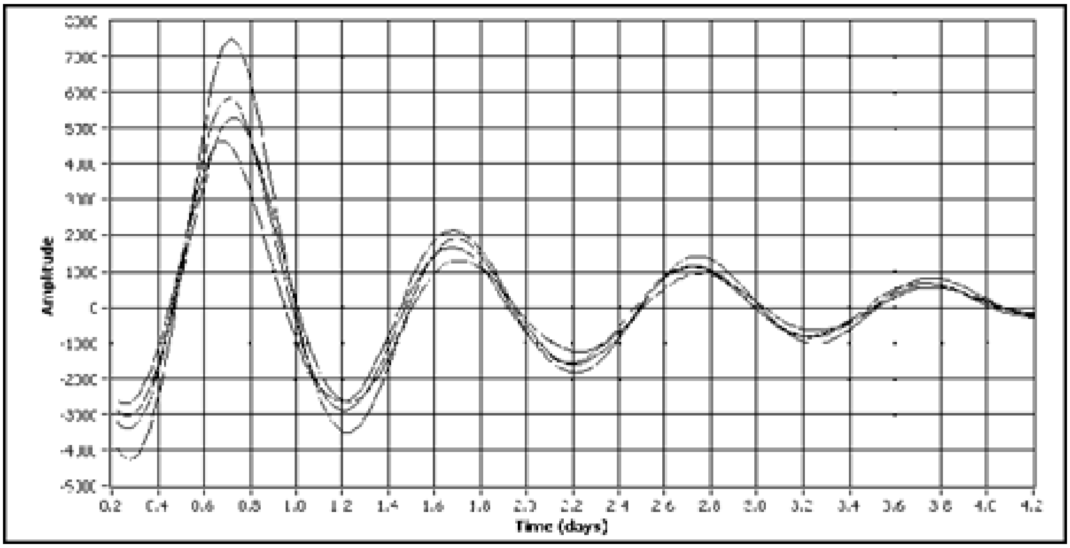


Synchronized $Nono^{gt}$

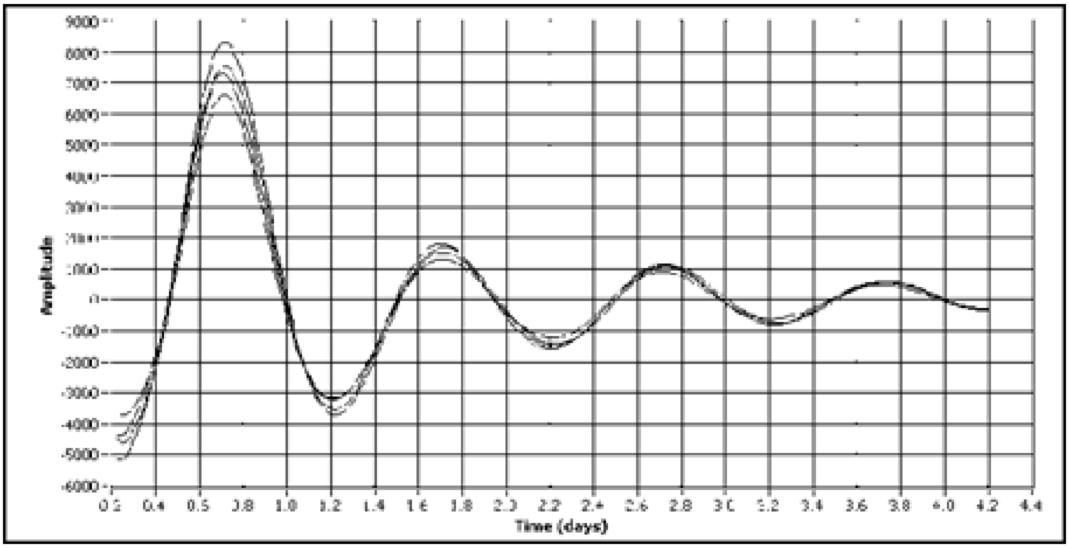


Unsynchronised WT

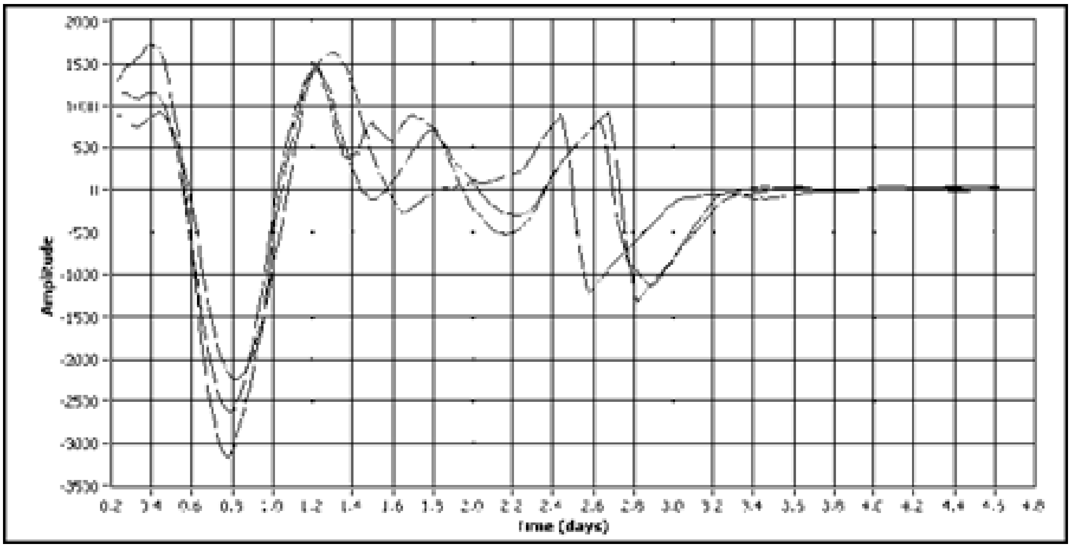

**Figure S1: control of the effective synchronization, related to STAR Methods section.**

Graphs showing the Real-Time Luminescence reporting of Bmal1-luciferase in WT and Nono$^{gt}$ MEFs, in three or four independent cell preparations (on the Y axis the luminescence amplitude, on the X axis the days from starting the measurement).

Only after Dexamethasone exposure the luminescence shows circadian oscillation lasting several days. In not synchronized cells, after a first oscillation due to the newly changed medium, only an almost flat line is detected. It must also be noted that the period does not change between WT and Nono$^{gt}$ MEFs (24.1 and 23.95 hours, respectively, calculated by the LumiCycle software from Actimetrics).

## Figure S2

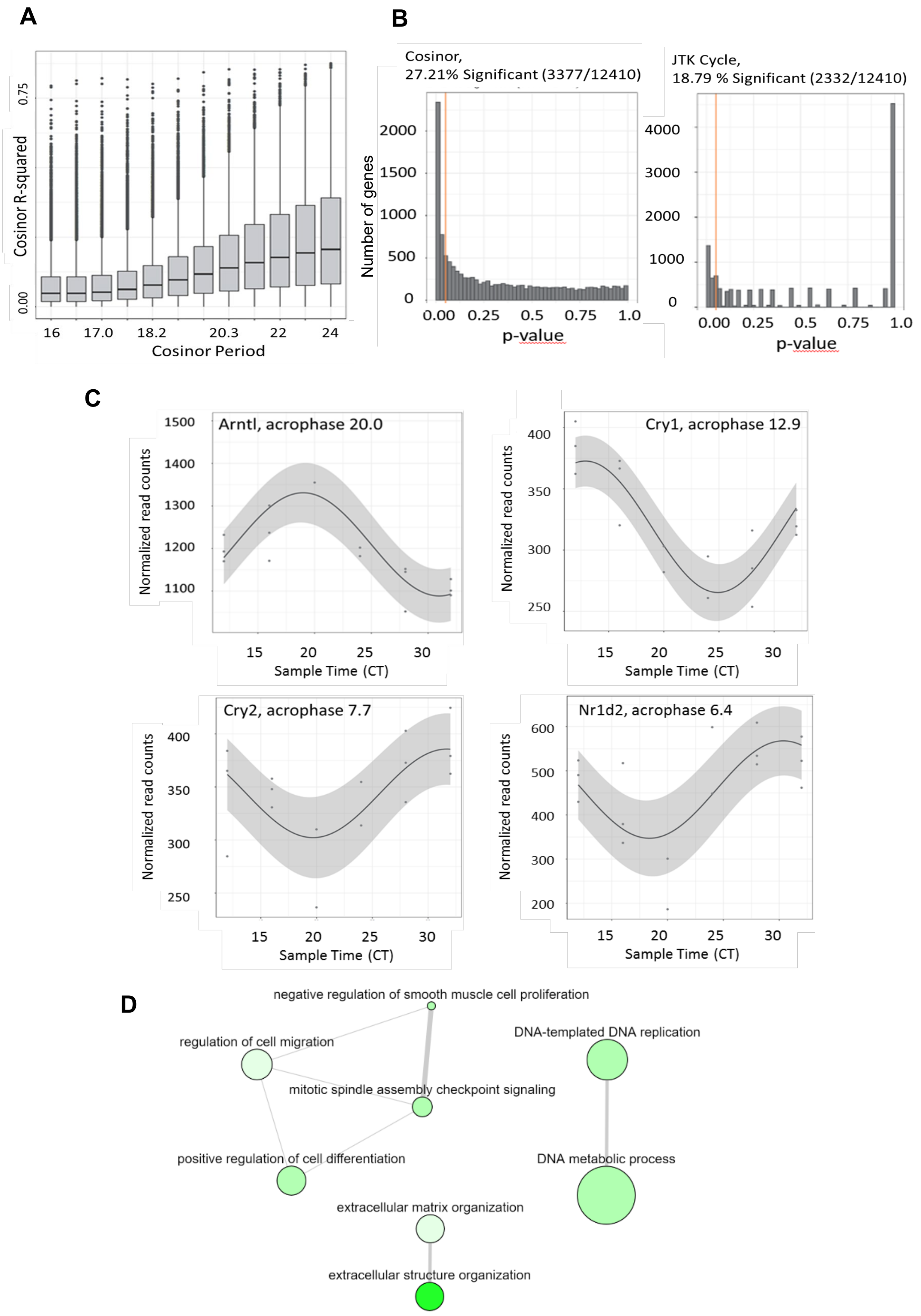
A
Cosinor R-squared
0.00
0.75
16
17.0
18.2
20.3
22
24
Cosinor Period
B
Cosinor,
27.21% Significant (3377/12410)
Number of genes
0
500
1000
1500
2000
0.00
0.25
0.5
0.75
1.0
p-value
JTK Cycle,
18.79 % Significant (2332/12410)
0
1000
2000
3000
4000
0.00
0.25
0.5
0.75
1.0
p-value
C
Arntl, acrophase 20.0
Normalized read counts
1100
1200
1300
1400
1500
15
20
25
30
Sample Time (CT)
Cry1, acrophase 12.9
Normalized read counts
250
300
350
400
15
20
25
30
Sample Time (CT)
Cry2, acrophase 7.7
Normalized read counts
250
300
350
400
15
20
25
30
Sample Time (CT)
Nr1d2, acrophase 6.4
Normalized read counts
200
300
400
500
600
15
20
25
30
Sample Time (CT)
D
negative regulation of smooth muscle cell proliferation
regulation of cell migration
DNA-templated DNA replication
mitotic spindle assembly checkpoint signaling
positive regulation of cell differentiation
DNA metabolic process
extracellular matrix organization
extracellular structure organization

**Figure S2: The circadian transcriptome in MEFs, related to Figure 1A and 1B.**

**(A)** Periodogram identifying the dominant periods and dominant cyclical behavior of the time series. **(B)** Histograms showing the number of the circadian genes identified by the Cosinor and JTK methods in MEFS ($p<0.05$). The corresponding percentage of total expressed genes is indicated for each method. **(C)** Example of Cosinor plots for the core clock genes with their acrophase. Cosinor analyses were performed on the RNA-seq data sets (time points from 12 to 32 hours after cell synchronization, 4-hours interval). **(D)** Gene ontology analysis of the enriched biological processes among circadian genes. A marked proportion of the rhythmic genes were involved in the regulation of mitotic steps and DNA replication, together with cell differentiation, migration and extracellular matrix organization. Normalized read counts from RNA-seq were analyzed using ShinyGO (expressed genes as background). Enrichment analysis is calculated based on hypergeometric distribution followed by a false discovery rate (FDR) correction, with the FDR cutoff value 0.05. The GO terms were then summarized by Revigo. The GO terms are represented by bubbles. The color intensity of the bubbles corresponds to the adjusted p-value (dark green for lower p-adj. value). The size of the bubble corresponds to the LogSize value of the GO term.

# Figure S3

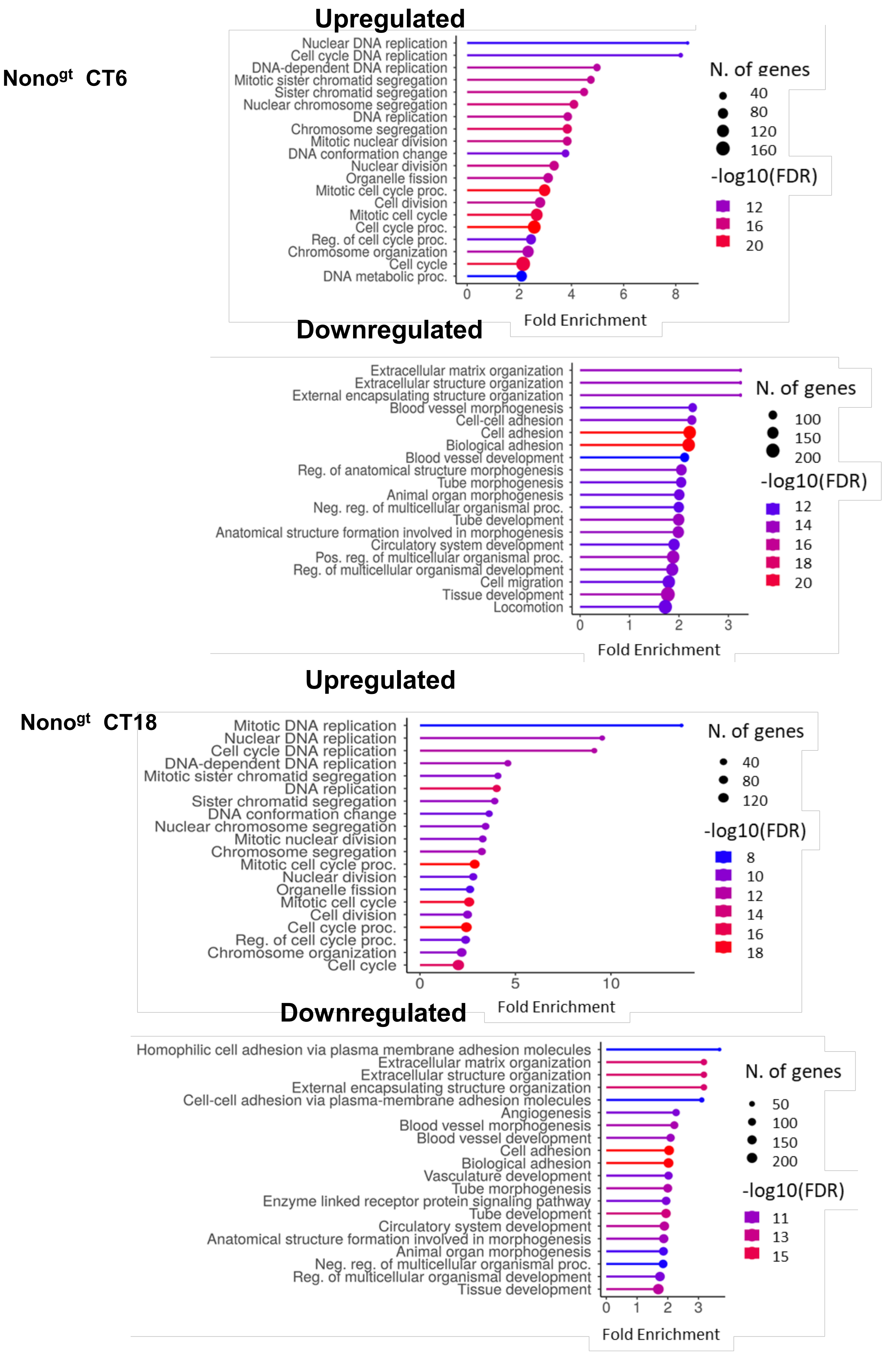

Nono^gt CT6
Upregulated
Nuclear DNA replication
Cell cycle DNA replication
DNA-dependent DNA replication
Mitotic sister chromatid segregation
Sister chromatid segregation
Nuclear chromosome segregation
DNA replication
Chromosome segregation
Mitotic nuclear division
DNA conformation change
Nuclear division
Organelle fission
Mitotic cell cycle proc.
Cell division
Mitotic cell cycle
Cell cycle proc.
Reg. of cell cycle proc.
Chromosome organization
Cell cycle
DNA metabolic proc.
0 2 4 6 8
Fold Enrichment
N. of genes
40
80
120
160
-log10(FDR)
12
16
20
Downregulated
Extracellular matrix organization
Extracellular structure organization
External encapsulating structure organization
Blood vessel morphogenesis
Cell-cell adhesion
Cell adhesion
Biological adhesion
Blood vessel development
Reg. of anatomical structure morphogenesis
Tube morphogenesis
Animal organ morphogenesis
Neg. reg. of multicellular organismal proc.
Tube development
Anatomical structure formation involved in morphogenesis
Circulatory system development
Pos. reg. of multicellular organismal proc.
Reg. of multicellular organismal development
Cell migration
Tissue development
Locomotion
0 1 2 3
Fold Enrichment
N. of genes
100
150
200
-log10(FDR)
12
14
16
18
20
Nono^gt CT18
Upregulated
Mitotic DNA replication
Nuclear DNA replication
Cell cycle DNA replication
DNA-dependent DNA replication
Mitotic sister chromatid segregation
DNA replication
Sister chromatid segregation
DNA conformation change
Nuclear chromosome segregation
Mitotic nuclear division
Chromosome segregation
Mitotic cell cycle proc.
Nuclear division
Organelle fission
Mitotic cell cycle
Cell division
Cell cycle proc.
Reg. of cell cycle proc.
Chromosome organization
Cell cycle
0 5 10
Fold Enrichment
N. of genes
40
80
120
-log10(FDR)
8
10
12
14
16
18
Downregulated
Homophilic cell adhesion via plasma membrane adhesion molecules
Extracellular matrix organization
Extracellular structure organization
External encapsulating structure organization
Cell-cell adhesion via plasma-membrane adhesion molecules
Angiogenesis
Blood vessel morphogenesis
Blood vessel development
Cell adhesion
Biological adhesion
Vasculature development
Tube morphogenesis
Enzyme linked receptor protein signaling pathway
Tube development
Circulatory system development
Anatomical structure formation involved in morphogenesis
Animal organ morphogenesis
Neg. reg. of multicellular organismal proc.
Reg. of multicellular organismal development
Tissue development
0 1 2 3
Fold Enrichment
N. of genes
50
100
150
200
-log10(FDR)
11
13
15

**Figure S3: Gene ontology analysis of Nono regulated genes, related to Figure 1C and 1D.**

Overrepresented gene ontology (GO) biological processes of the differentially expressed genes in Nono$^{gt}$ MEFs when compared to WT controls. Analysis and visualization were carried out using ShinyGO (expressed genes as background). Enrichment analysis is calculated based on hypergeometric distribution followed by a false discovery rate (FDR) correction. Overrepresented GO terms are selected by FDR (cutoff < 0.05) and ranked by fold enrichment.

# Figure S4

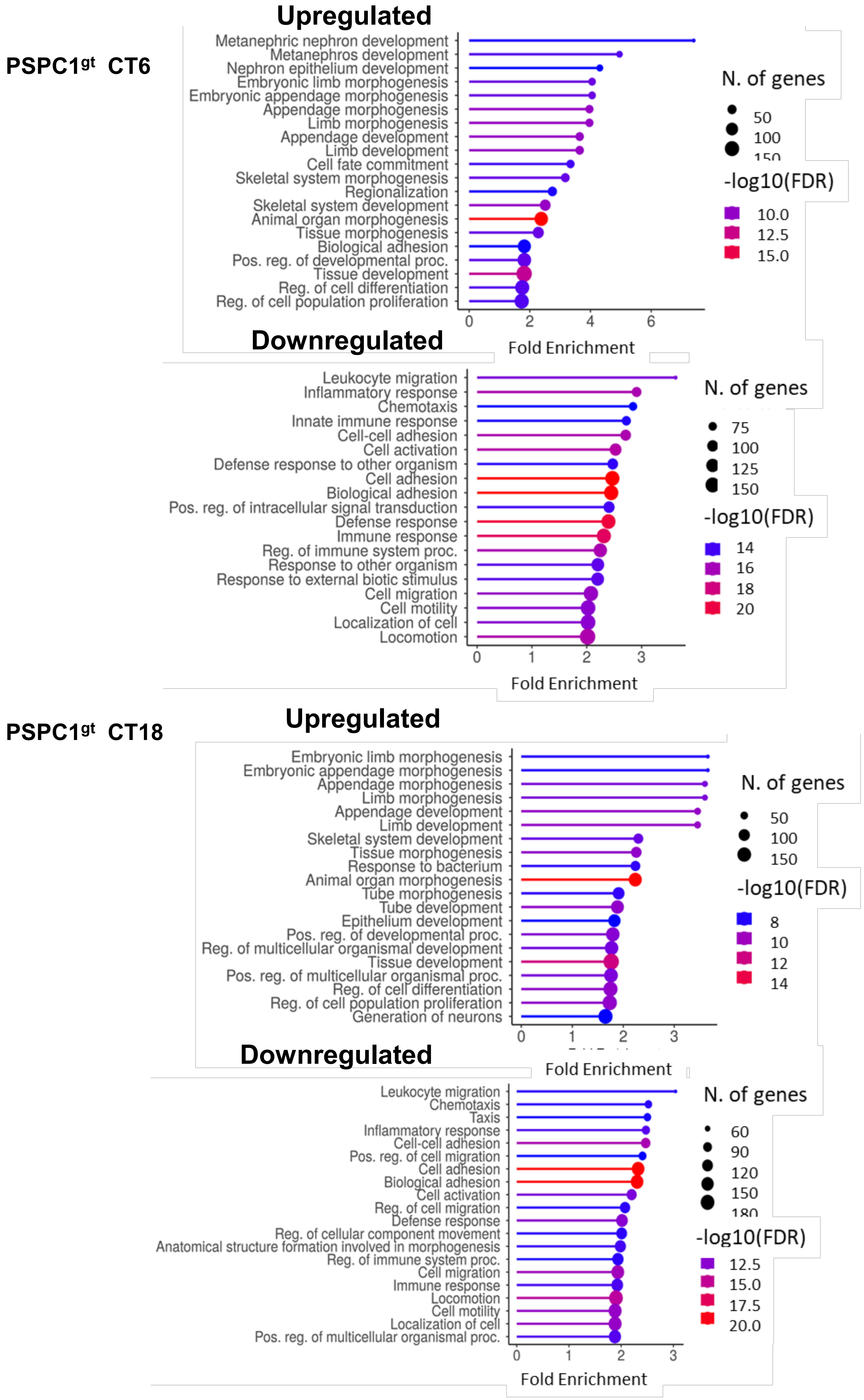
PSPC1gt CT6
Upregulated
Metanephric nephron development
Metanephros development
Nephron epithelium development
Embryonic limb morphogenesis
Embryonic appendage morphogenesis
Appendage morphogenesis
Limb morphogenesis
Appendage development
Limb development
Cell fate commitment
Skeletal system morphogenesis
Regionalization
Skeletal system development
Animal organ morphogenesis
Tissue morphogenesis
Biological adhesion
Pos. reg. of developmental proc.
Tissue development
Reg. of cell differentiation
Reg. of cell population proliferation
0 2 4 6
Fold Enrichment
N. of genes
50
100
150
-log10(FDR)
10.0
12.5
15.0
Downregulated
Leukocyte migration
Inflammatory response
Chemotaxis
Innate immune response
Cell-cell adhesion
Cell activation
Defense response to other organism
Cell adhesion
Biological adhesion
Pos. reg. of intracellular signal transduction
Defense response
Immune response
Reg. of immune system proc.
Response to other organism
Response to external biotic stimulus
Cell migration
Cell motility
Localization of cell
Locomotion
0 1 2 3
Fold Enrichment
N. of genes
75
100
125
150
-log10(FDR)
14
16
18
20
PSPC1gt CT18
Upregulated
Embryonic limb morphogenesis
Embryonic appendage morphogenesis
Appendage morphogenesis
Limb morphogenesis
Appendage development
Limb development
Skeletal system development
Tissue morphogenesis
Response to bacterium
Animal organ morphogenesis
Tube morphogenesis
Tube development
Epithelium development
Pos. reg. of developmental proc.
Reg. of multicellular organismal development
Tissue development
Pos. reg. of multicellular organismal proc.
Reg. of cell differentiation
Reg. of cell population proliferation
Generation of neurons
0 1 2 3
Fold Enrichment
N. of genes
50
100
150
-log10(FDR)
8
10
12
14
Downregulated
Leukocyte migration
Chemotaxis
Taxis
Inflammatory response
Cell-cell adhesion
Pos. reg. of cell migration
Cell adhesion
Biological adhesion
Cell activation
Reg. of cell migration
Defense response
Reg. of cellular component movement
Anatomical structure formation involved in morphogenesis
Reg. of immune system proc.
Cell migration
Immune response
Locomotion
Cell motility
Localization of cell
Pos. reg. of multicellular organismal proc.
0 1 2 3
Fold Enrichment
N. of genes
60
90
120
150
180
-log10(FDR)
12.5
15.0
17.5
20.0

**Figure S4: Gene ontology analysis of PSPC1 regulated genes, related to Figure 1E and 1F.**

Overrepresented gene ontology (GO) biological processes of the differentially expressed genes in PSPC1$^{gt}$ MEFs when compared to WT controls. Analysis and visualization are as in **Figure S3**.

## Figure S5

A

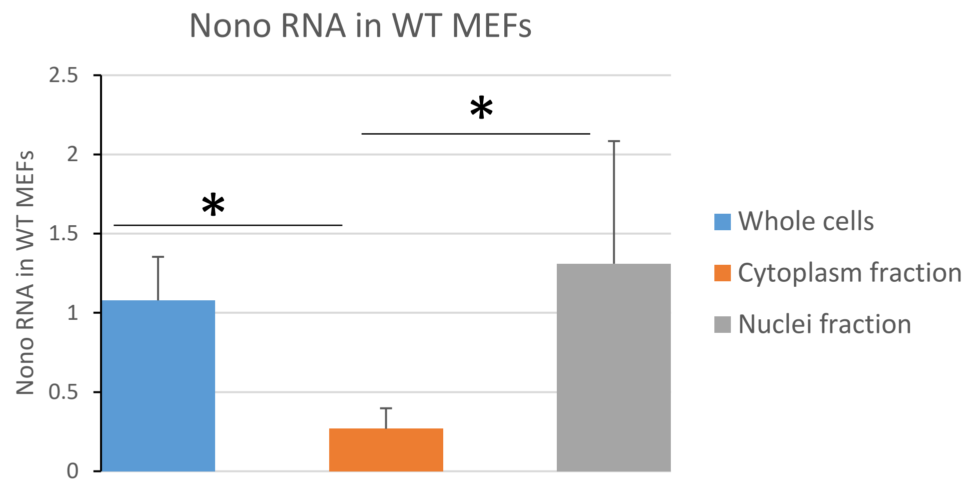


B

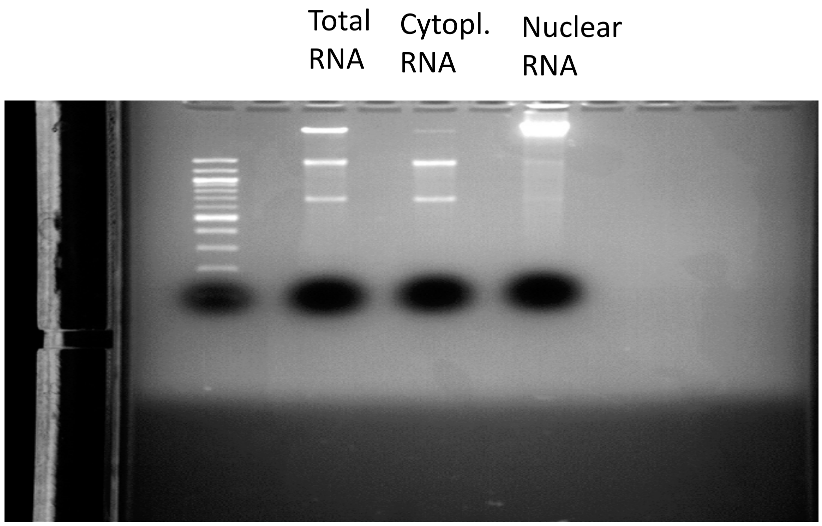


C

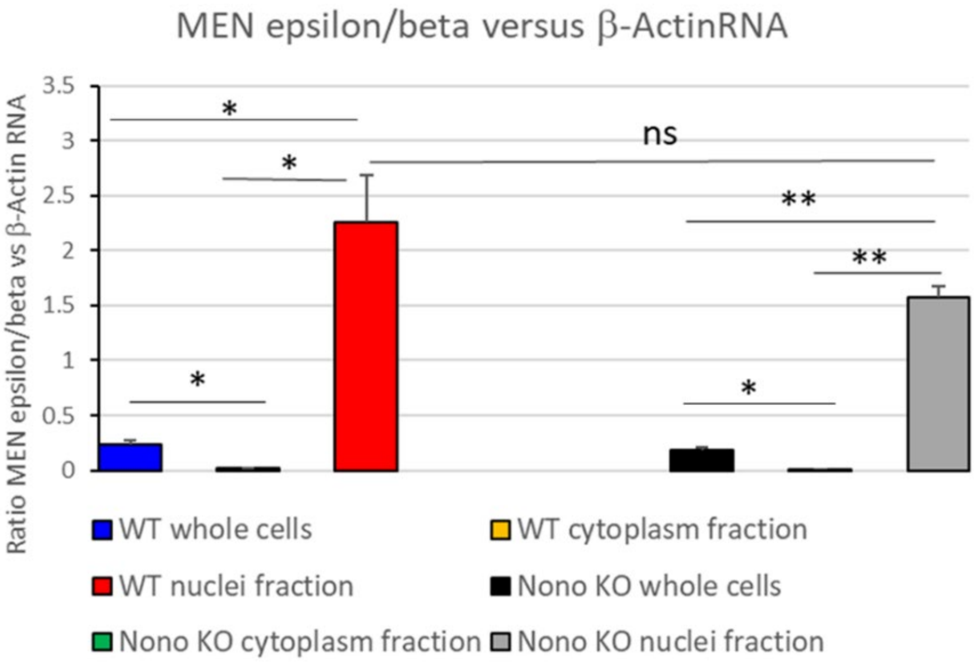

**Figure S5: Validation of the cytoplasm and nuclei separation, related to the STAR Methods section.**

**(A)** Bar graph showing the ratio of Nono RNA in the cytoplasmic and nuclear fractions of WT MEFs vs the whole cells. Data are presented as mean ± SEM. Unpaired t-test, * p-value < 0.05. As expected, Nono RNA is much less abundant in the cytoplasm compared to the nucleus or whole cell.

**(B)** Agarose gel of RNA from whole cells, cytoplasmic and nuclear fractions. 0.5 µg of RNA from total cells, cytoplasmic fractions and nuclear fraction of the MEFs were evaluated in agarose gel 1.5%. The RNA appeared intact, with the rRNA bands well evident in the total and in the cytoplasmic RNA and only very faint in the nuclear RNA.

**(C)** Bar graph showing the ratio between the Men-epsilon/beta RNA (nuclear-retained non-coding RNAs, as nuclear marker) and the beta-Actin RNA (the main component of the cytoskeleton, as cytoplasmic marker) in the whole cells and after cytoplasm and nuclei separation in WT and $Nono^{gt}$ MEFs, obtained by qPCR, n=3. Data are presented as mean ± SEM. Unpaired t-test, * p-value < 0.05, ** p-value < 0.01. The Men epsilon/beta RNA makes up about 20% of the β-Actin RNA in whole cells. In the cytoplasm the β-Actin RNA was extremely more abundant than Men epsilon beta RNA (about hundred folds), whereas in the nuclei fraction the Men epsilon/beta was more abundant. That validates the cell fractionation method.

# Figure S6

**A**

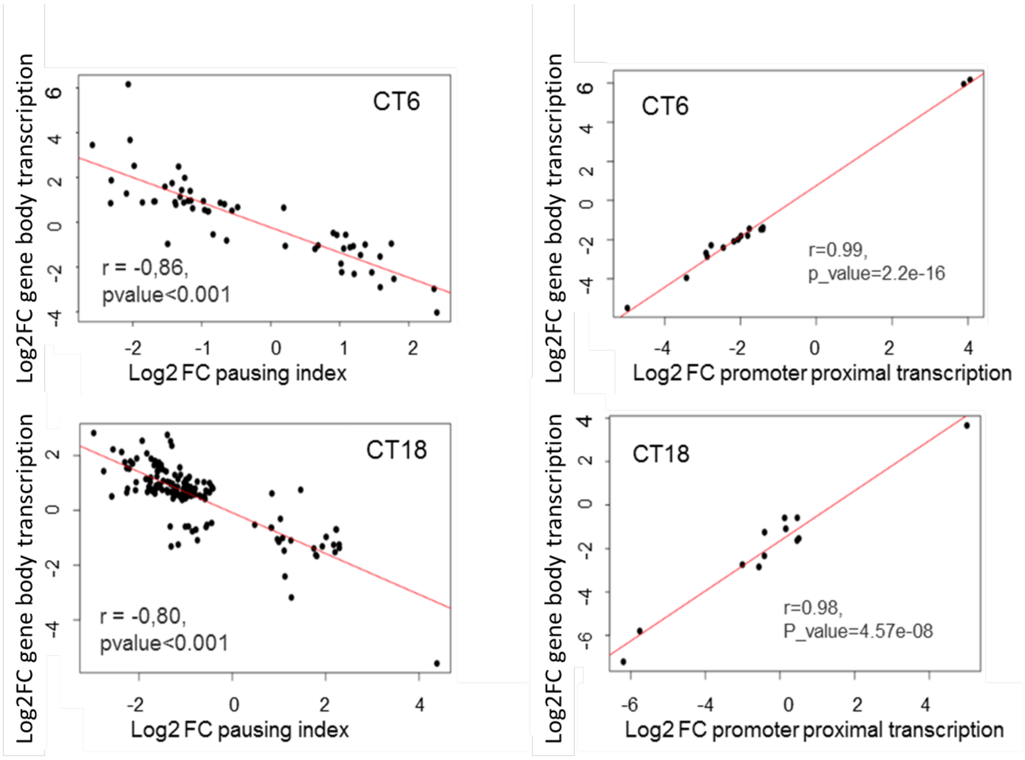

CT6
r = -0,86,
pvalue<0.001
Log2 FC pausing index
Log2FC gene body transcription
CT6
r=0.99,
p_value=2.2e-16
Log2 FC promoter proximal transcription
Log2FC gene body transcription
CT18
r = -0,80,
pvalue<0.001
Log2 FC pausing index
Log2FC gene body transcription
CT18
r=0.98,
P_value=4.57e-08
Log2 FC promoter proximal transcription
Log2FC gene body transcription


**B**

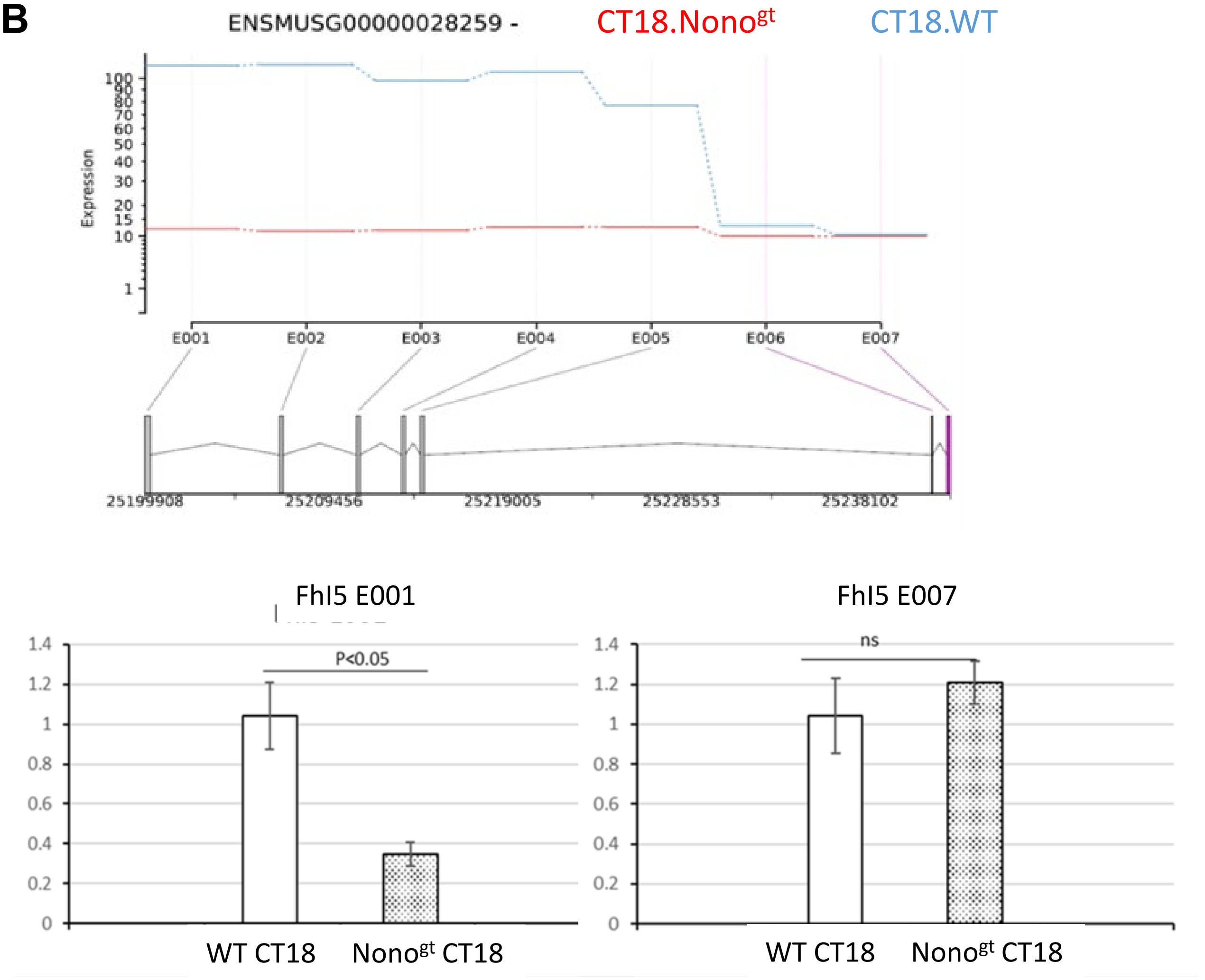

ENSMUSG00000028259 -
CT18.Nono$^{gt}$
CT18.WT
Expression
E001
E002
E003
E004
E005
E006
E007
25199908
25209456
25219005
25228553
25238102
Fhl5 E001
P<0.05
WT CT18
Nono$^{gt}$ CT18
Fhl5 E007
ns
WT CT18
Nono$^{gt}$ CT18

**Figure S6: assays validation of GRO-Seq and Differential Exons Usage, related to Figure 3C, 3D and the STAR Methods section.**

**(A)** GRO-Seq**.** Left side: scatter plot showing the negative correlation between the pausing of polymerase immediately downstream of a transcription start site (56 genes out of 261 at CT6, 146 genes out of 408 at CT18) and the gene body transcription. Right side: scatter plot showing the positive correlation between the polymerase density in the promoter-proximal regions (16 genes out of 18 at CT6, 12 out of 16 at CT18) and the transcription of gene bodies. The sample correlation coefficients and p values are shown in the plots. **(B)** Differential exon usage. Up: Graph from JunctionSeq of the exon usage of Fhl5 (Four And A Half LIM Domains Protein 5) gene. Down: expression levels of exon 1 and exon 7, obtained by qPCR, n=3. Data are presented as mean ± SEM. Unpaired t-test, $p$-value $< 0.05$.

# Figure S7

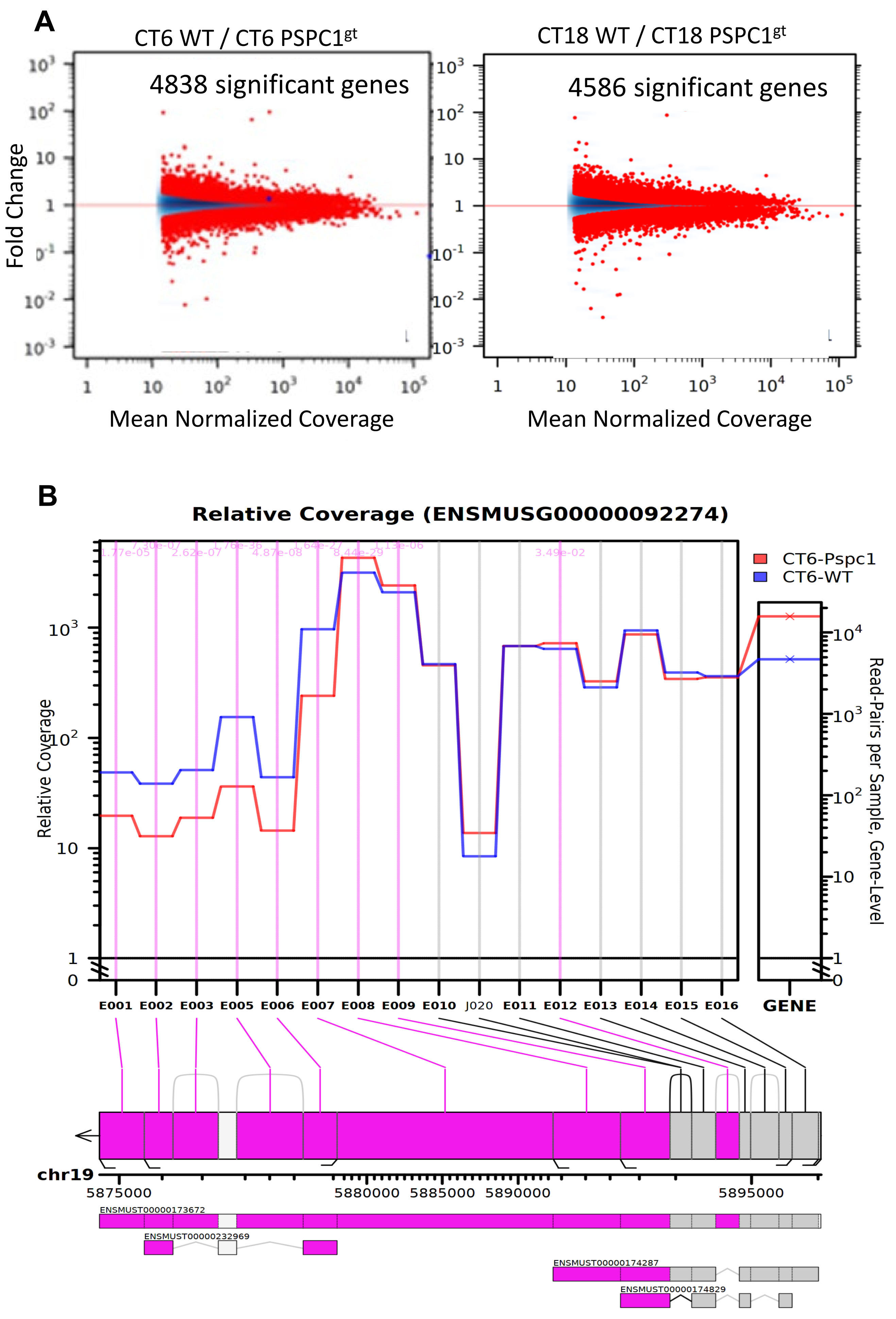

A
CT6 WT / CT6 PSPC1gt
4838 significant genes
CT18 WT / CT18 PSPC1gt
4586 significant genes
Fold Change
Mean Normalized Coverage
Mean Normalized Coverage
B
Relative Coverage (ENSMUSG00000092274)
CT6-Pspc1
CT6-WT
Relative Coverage
Read-Pairs per Sample, Gene-Level
E001 E002 E003 E005 E006 E007 E008 E009 E010 J020 E011 E012 E013 E014 E015 E016
GENE
chr19
5875000
5880000 5885000 5890000
5895000
ENSMUST00000173672
ENSMUST00000232969
ENSMUST00000174287
ENSMUST00000174829

**Figure S7: PSPC1 regulation of splicing, related to Figure 4.**

**(A)** MA plots (i.e scatter plots of log fold changes versus the mean of normalized counts) showing the extent of differential usage of exons and splice junctions in PSPC1$^{gt}$ versus WT at CT6 and CT18. The number in the plot shows the analysis at gene-level for the hypothesis that one or more features belonging to this gene are differentially used (JuncionSeq software, GeneWise P adj. value < 0.05). **(B)** JunctionSeq graph showing relative read coverage across exon region of the *NEAT1* locus in WT and PSPC1$^{gt}$. The gene-wide expression is plotted in the right panel. The exonic regions being statistically significant (FDR<0.01) are marked with vertical pink lines. The annotated transcripts are displayed below the plot.

# Figure S8

CT6

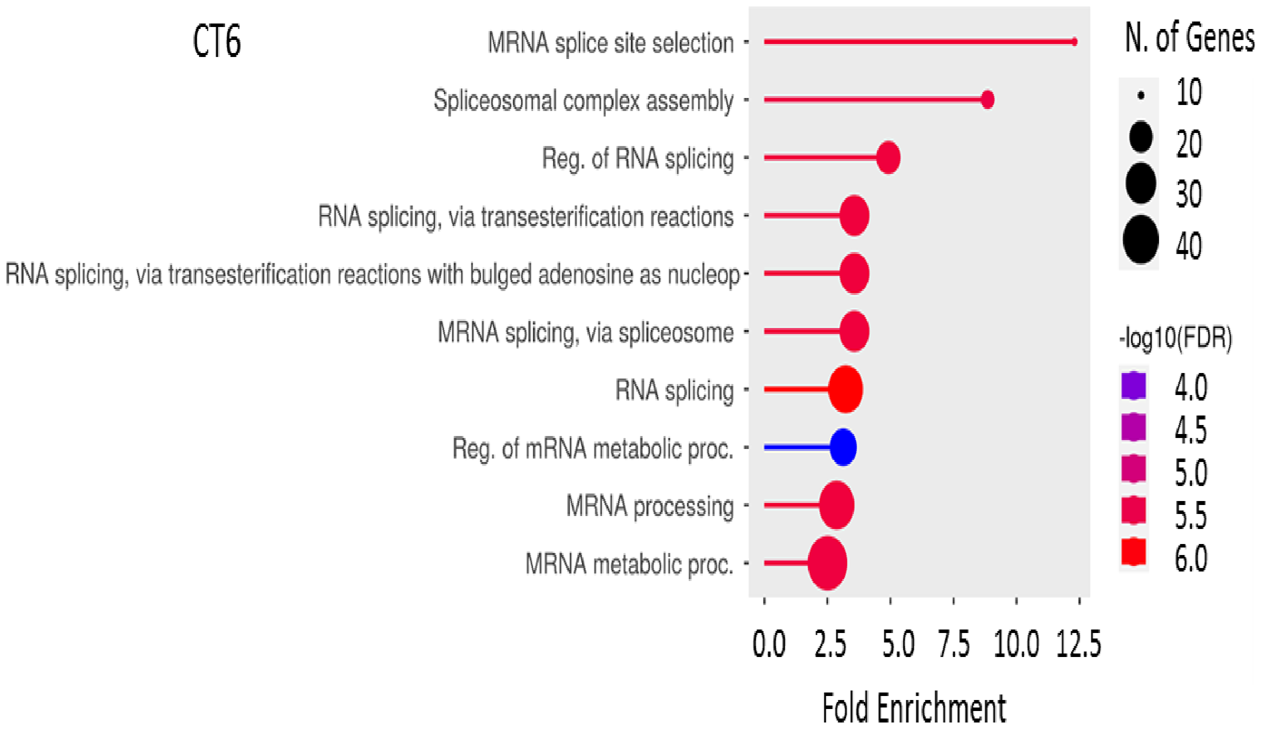


CT18

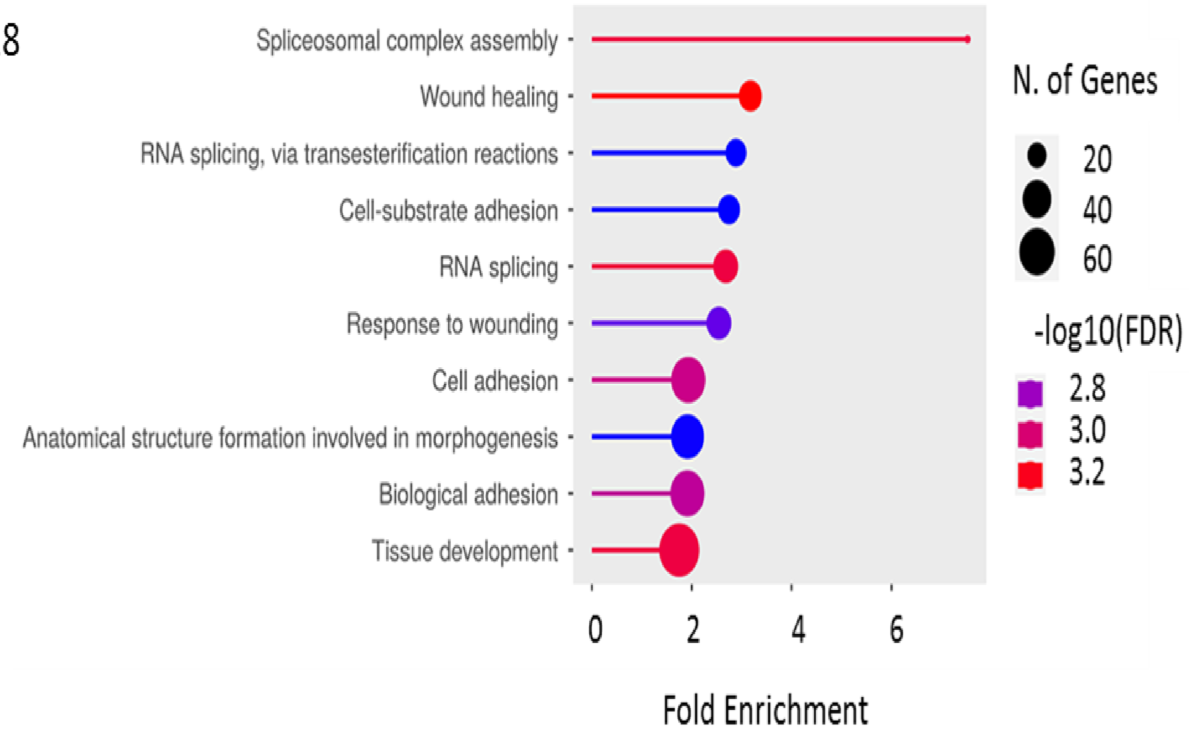

**Figure S8: Nono depletion alters alternative splicing and spliceosome assembly, related to Figure 4.**

Gene ontology analysis of the genes showing differential splicing in $Nono^{gt}$ compared to WT cells at the indicated time point. Analysis and visualization were carried out using ShinyGO (expressed genes as background). Enrichment analysis is calculated based on hypergeometric distribution followed by a false discovery rate (FDR) correction. Overrepresented GO terms are selected by FDR (cutoff < 0.05) and ranked by fold enrichment.

## Figure S9

**A**

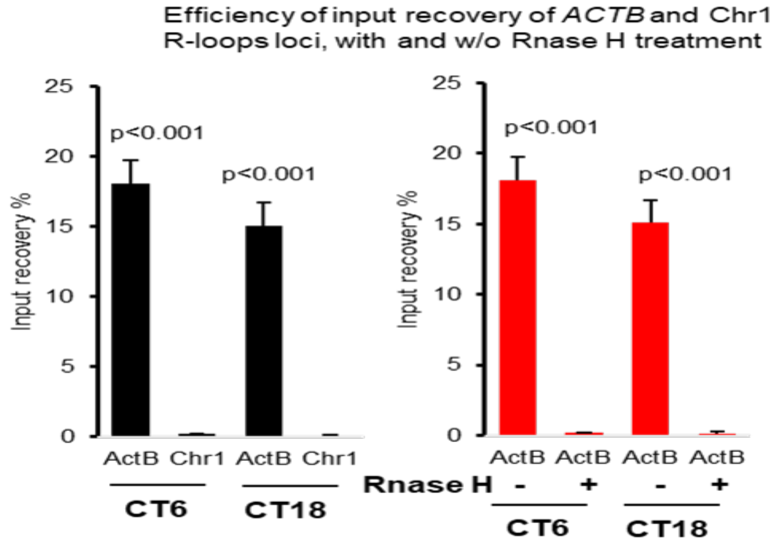


**B** DRIP-seq read distribution along gene

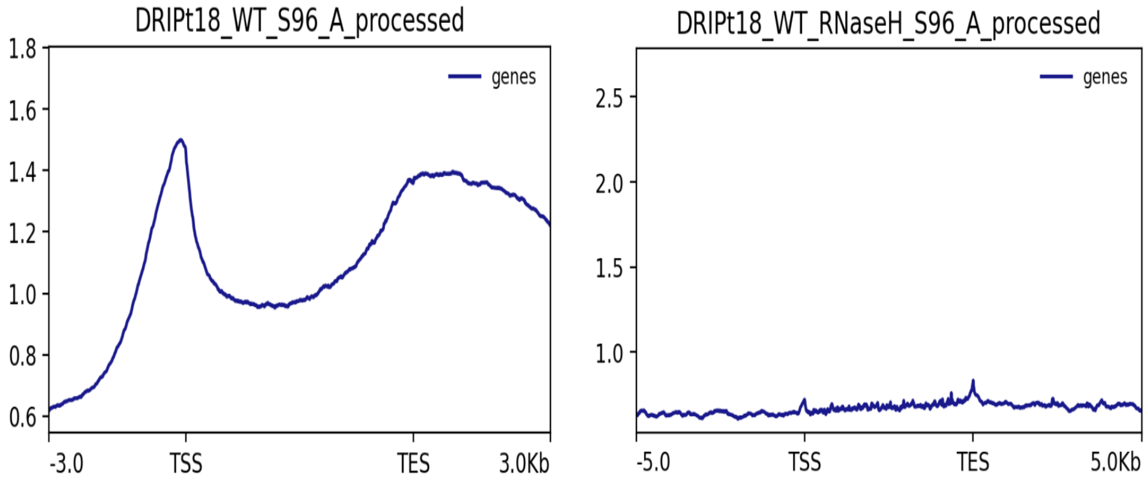


**C**

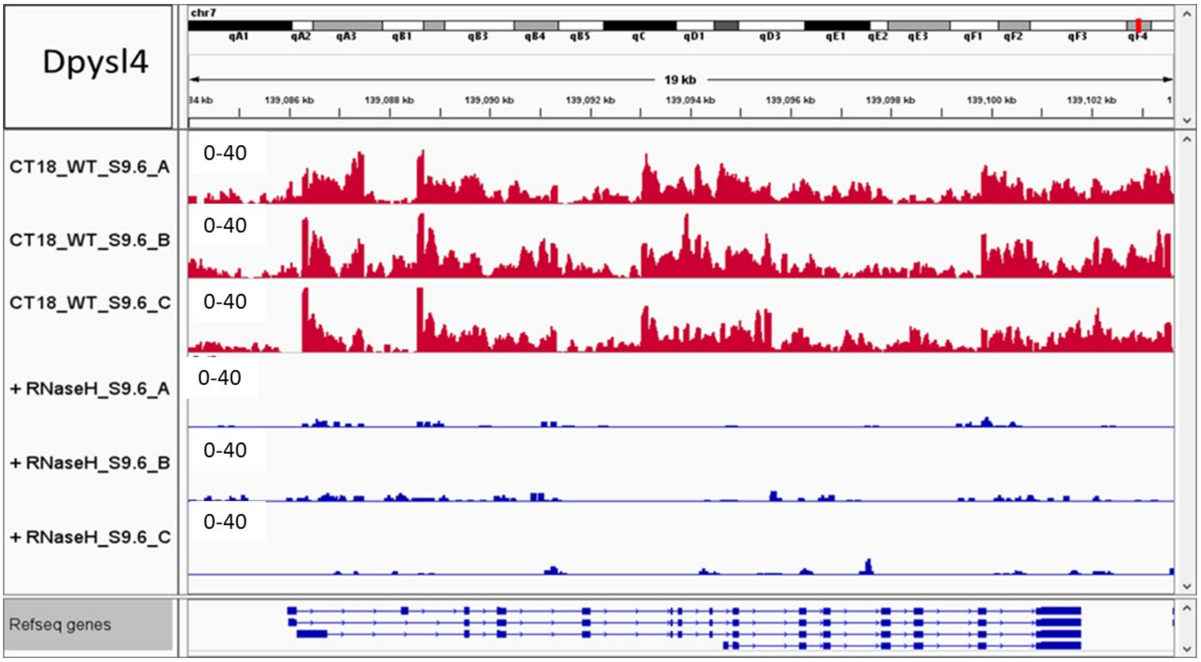

**Figure S9**: **Experimental validation and specificity of the R-loops detection by DRIP, related to the STAR Methods section.**

DRIP samples were analyzed by real-time PCR, n=3. **(A)** Bar graph showing the R-loops enrichment within the *ACTB* gene locus (a specific region with R-loops presence). A Chr1 locus site devoid of R-loops as well as RNase H treatment were used to check the specificity of DRIP. Data are presented as mean ± SEM. Unpaired t-test, p-value < 0.001. Note the high input recovery for the ACTB region, that dramatically fell when the samples were treated with RNase H.

**(B)** Profile plot showing an example of the DRIP-seq reads distribution along the gene, before normalization. All reads are used, including intergenic ones. The reads enrichment, localized to the TSS/promoter and to TTS, was completely abolished after RNase H treatment, confirming the specificity of the detected peaks**. (C)** Visualization of R-loops enrichment across the *Dpysl4* locus in S9.6 antibody IP samples, with and without pretreatment with RNase H, using Integrated Genome Viewer (IGV).

# Figure S10

**A**

Differential expression of genes with R-loops gain and loss, at the indicated times

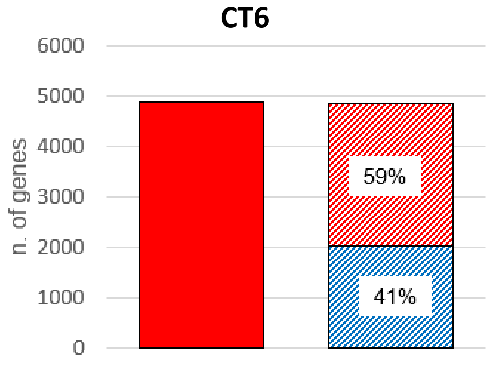


Correlation of R-loops gain with RNA-seq FC:
Pearson correlation coefficient r=0.07
P-value 1.61e-6

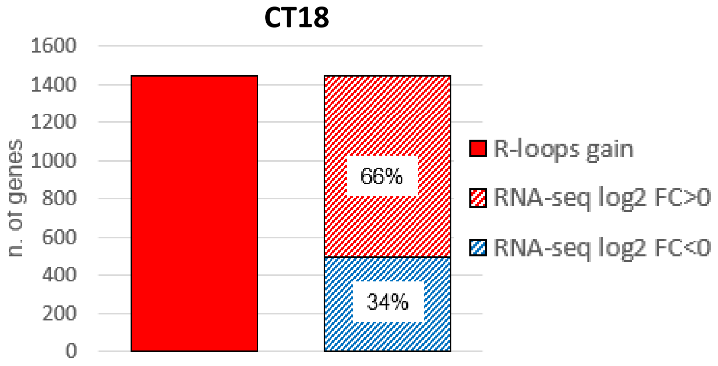


Correlation of R-loops gain with RNA-seq FC:
Pearson correlation coefficient r=0.05
P-value: 0.037

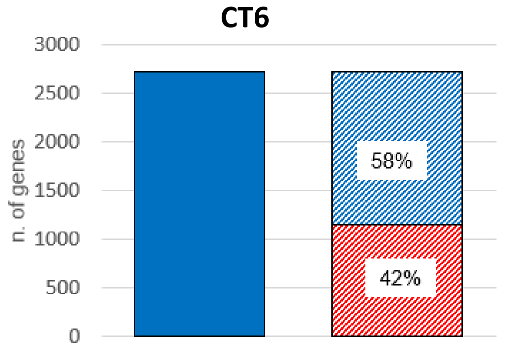


Correlation of R-loops loss with RNA-seq FC:
Pearson correlation coefficient r=0.12
P-value: 1.57e-11

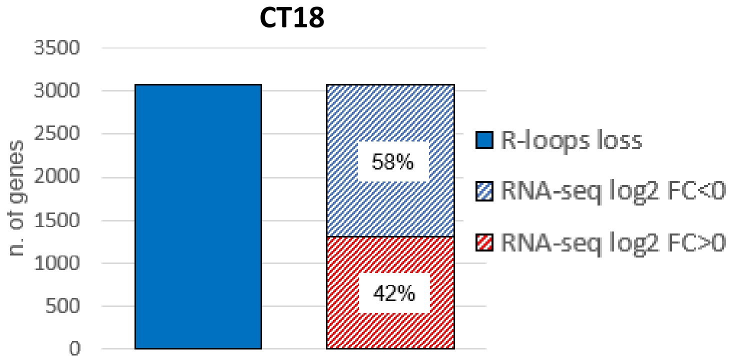


Correlation of R-loops loss with RNA-seq FC:
Pearson correlation coefficient (r=0.14)
P-value 1.78e-15

**B**

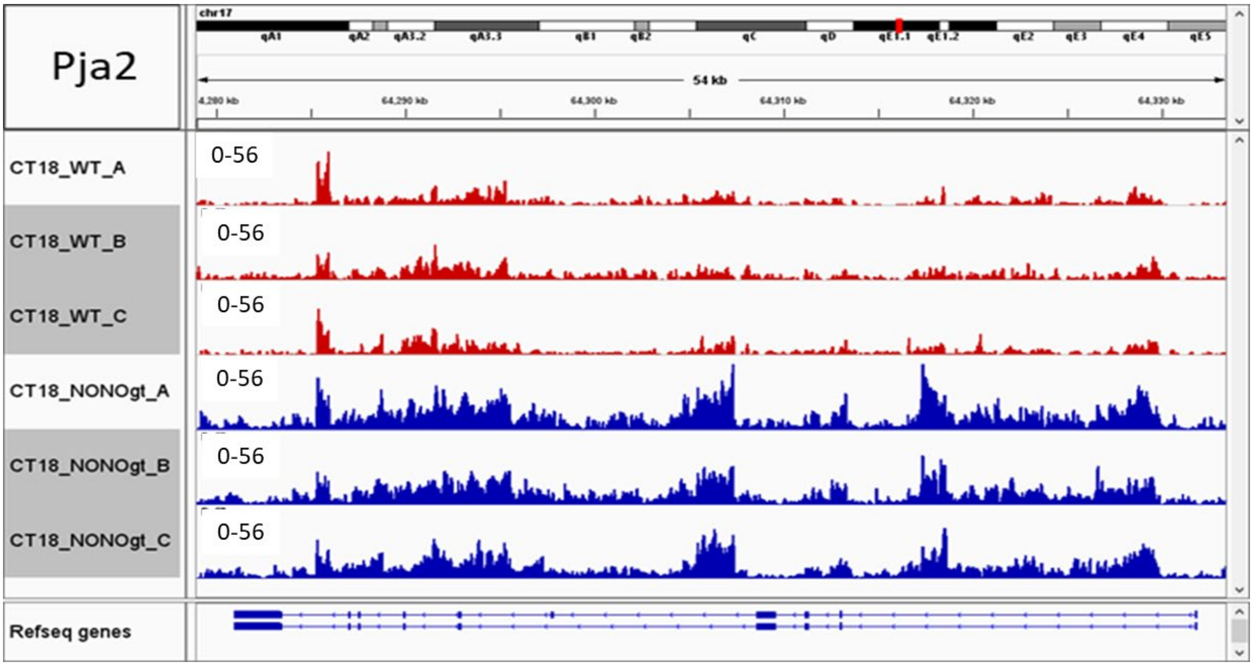

**Figure S10: Transcriptional variations do not account for the R-loops gain and loss in Nono$^{gt}$ versus WT MEFs, related to Figure 5B, Figure 1C, and Tables S2-S3.**

**(A)** Graph showing the variation of gene expression as a function of R-loops gain or loss, at the indicated time points. Below, the graph coefficient *r* of the Pearson correlation analysis of the two variables is shown. Following Cohen's guidelines (Cohen J., 1988, Statistical power analysis for the behavioral sciences, 2nd ed., p.413) [42]:

$|r| < 0.1$ Very small correlation effect size

$0.1 \le |r| < 0.3$ small correlation effect size,

the linear Pearson correlation analysis indicated a small/very small linear relationship between the two variables.

**(B)** Integrative Genomics Viewer (IGV) snapshot depicting R-loops gain or loss across the *Pja2* locus at CT18. Note that Pja2 is not differentially expressed between the two genotypes (Nono$^{gt}$ versus WT: log2FC -0.2, from RNA-seq) while in Nono$^{gt}$ R-loops peaks gain was observed.

**Figure S11**

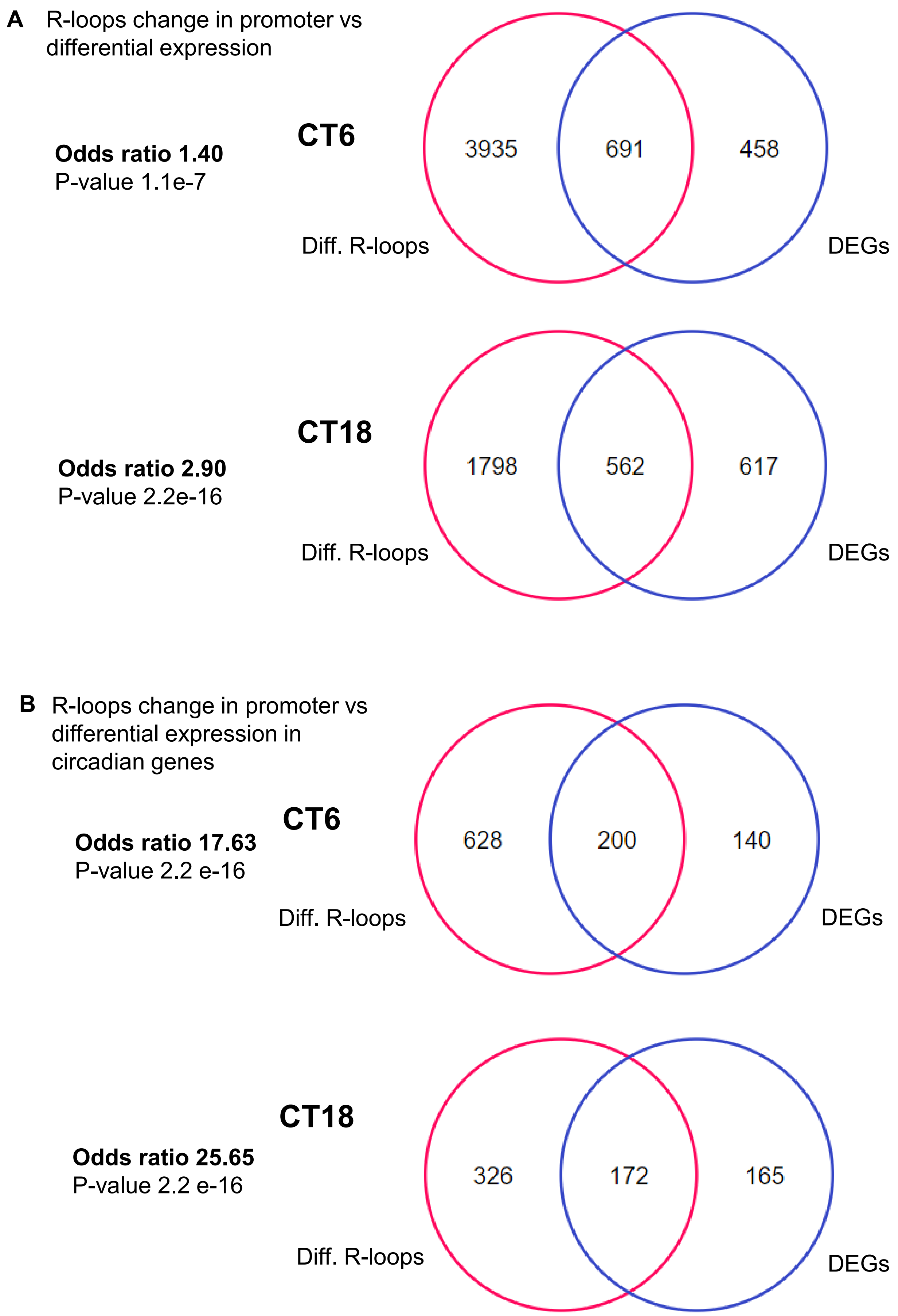
A
R-loops change in promoter vs differential expression
CT6
Odds ratio 1.40
P-value 1.1e-7
3935
691
458
Diff. R-loops
DEGs
CT18
Odds ratio 2.90
P-value 2.2e-16
1798
562
617
Diff. R-loops
DEGs
B
R-loops change in promoter vs differential expression in circadian genes
CT6
Odds ratio 17.63
P-value 2.2 e-16
628
200
140
Diff. R-loops
DEGs
CT18
Odds ratio 25.65
P-value 2.2 e-16
326
172
165
Diff. R-loops
DEGs

**Figure S11: Functional association of R-loops in the promoters of circadian genes with their expression, related to Figure 5C.**

**(A)** Genes with R-loops in the promoter: Venn diagrams showing the overlap between the changes of R-loops in the promoter region and the differential expression from RNA-seq, at the indicated time points (Fisher exact test: CT6 odds ratio 1.40, p-value 1.1e-7; CT18: odds ratio 2.90, p-value 2.2e-16). **(B)** Circadian genes with R-loops in the promoter: Venn diagram of the significant overlap between the presence of R-loops in the promoter region and their differential expression from RNA-seq, at the indicated time points (Fisher exact test: CT6 odds ratio 17.63, p-value 2.2e-16; CT18: odds ratio 25.65, p-value 2.2e-16).

# Figure S12

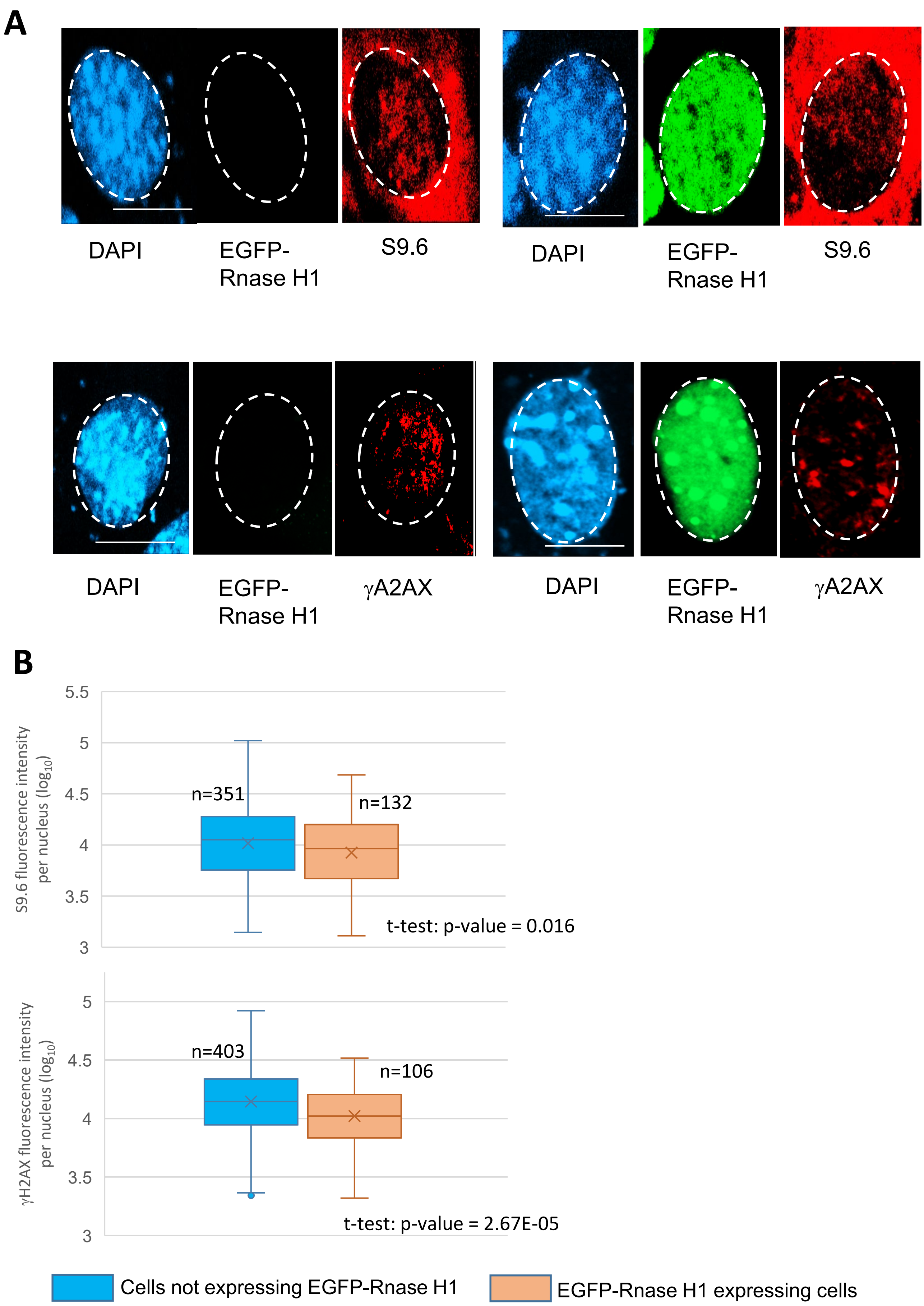

A
DAPI
EGFP-
Rnase H1
S9.6
DAPI
EGFP-
Rnase H1
S9.6
DAPI
EGFP-
Rnase H1
γA2AX
DAPI
EGFP-
Rnase H1
γA2AX
B
S9.6 fluorescence intensity per nucleus (log10)
5.5
5
4.5
4
3.5
3
n=351
n=132
t-test: p-value = 0.016
γH2AX fluorescence intensity per nucleus (log10)
5
4.5
4
3.5
3
n=403
n=106
t-test: p-value = 2.67E-05
Cells not expressing EGFP-Rnase H1
EGFP-Rnase H1 expressing cells

**Figure S12: Transient nuclear expression of RNase H1 reduces R-loops accumulation and DNA damage, related to Fig. 7 and the STAR Methods section.** To confirm that the DNA damage induced by BRD4 inhibition is R-loops dependent, in a side experiment we transfected WT MEFs with a plasmid for the transient mammalian expression of wild type RNase H1, tagged with 2xNLS and EGFP, in order to specifically degrade the nuclear R-loops. **(A)** Examples of nuclear S9.6 (anti-mouse Alexa fluor 594) (R-loops) and γH2AX (anti-rabbit Alexa fluor 594) (DNA damage) immunofluorescence intensity in WT cells successfully expressing EGFP-RNase H1 plasmid versus cells not expressing the EGFP. All the cells were exposed to ARV-825 30 nM for 6 hours. Scale bar 15 μM. **(B)** Box plots showing the analysis of S9.6 and γH2AX integrated fluorescence per nucleus in cells expressing EGP-RNAse H1 versus cells not expressing EGFP, all treated with ARV-825 30nM for 6 hours. The experiment was carried out in triplicate independent wells and the cells with and without EGFP expression were counted in the same fields. The data were log transformed to obtain normality (Shapiro-Wilk test) and equal variances (two-tailed F test), then the two-tailed unpaired t-test was applied. We observed a reduction of S9.6 fluorescence intensity per nucleus when RNase H1-EGP was expressed and a concomitant significant lower level of γH2AX signal, indicating the R-loops accumulation as responsible for the DNA damage after BRD4 inhibition.

**Supplementary Table 1**

| Oligonucleotides list: | Manufacturer | Sequence |
|---|---|---|
| | | |
| MEN epsilon/beta forward strand primer | Microsynth (CH) | 5’-GGGAAGGGTGACATTGAAAA-3’ |
| MEN epsilon/beta reverse strand primer | Microsynth (CH) | 5’-CTCCCCAGCTTCACTTCTTG-3’ |
| | | |
| beta-Actin forward strand primer | Microsynth (CH) | 5’-GGCTGTATTCCCCTCCATCG-3’ |
| beta-Actin reverse strand primer | Microsynth (CH) | 5’-CCAGTTGGTAACAATGCCATG-3’ |
| | | |
| Actin positive locus in DRIP, foward strand primer | Microsynth (CH) | 5'-TGCTCCCCGGGCTGTATT-3' |
| Actin positive locus in DRIP, reverse strand primer | Microsynth (CH) | 5'-ACATAGGAGTCCTTCTGACCCATT-3' |
| | | |
| Chr1 negative locus in DRIP, foward strand primer | Microsynth (CH) | 5'-TTCCAACAAAGCAGCAAATG-3' |
| Chr1 negative locus in DRIP, reverse strand primer | Microsynth (CH) | 5'-GGGTCACCAGACCTGTTTTT-3' |
| | | |
| Fhl5 Exon1 forward strand primer | Microsynth (CH) | 5'-ACT GCC GAC AGC CAA TAG GA-3' |
| Fhl5 Exon1 reverse strand primer | Microsynth (CH) | 5'-CTC CTT CTC AAA ACA TGG CAC AC-3' |
| | | |
| Fhl5 Exon7 reverse strand primer | Microsynth (CH) | 5'-ATCTGCTTTCAAGACCGCC-3' |
| Fhl5 Exon7 reverse strand primer | Microsynth (CH) | 5'-CTTCACCCACCAAGGAGACC-3' |
| | | |
| Nono forward strand primer | Microsynth (CH) | 5'-AACCAGGAGAGAAGACCTTTACACA-3' |
| Nono reverse strand primer | Microsynth (CH) | 5'-AAACTTCGCCTGCTTTTCCA-3' |
